# Amyloid β-peptides 1–40 and 1–42 form oligomers with mixed β-sheets†
†Electronic supplementary information (ESI) available. See DOI: 10.1039/c7sc01743j


**DOI:** 10.1039/c7sc01743j

**Published:** 2017-10-12

**Authors:** Maurizio Baldassarre, Cesare M. Baronio, Ludmilla A. Morozova-Roche, Andreas Barth

**Affiliations:** a Department of Biochemistry and Biophysics , Stockholm University , Arrhenius Laboratories , 10691 Stockholm , Sweden . Email: barth@dbb.su.se; b Department of Medical Biochemistry and Biophysics , Umeå University , 90187 Umeå , Sweden

## Abstract

Two main amyloid-β peptides of different length (Aβ_40_ and Aβ_42_) are involved in Alzheimer's disease. Their relative abundance is decisive for the severity of the disease and mixed oligomers may contribute to the toxic species. However, little is know about the extent of mixing. To study whether Aβ_40_ and Aβ_42_ co-aggregate, we used Fourier transform infrared spectroscopy in combination with ^13^C-labeling and spectrum calculation and focused on the amide I vibration, which is sensitive to backbone structure. Mixtures of monomeric labeled Aβ_40_ and unlabeled Aβ_42_ (and *vice versa*) were co-incubated for ∼20 min and their infrared spectrum recorded. The position of the main ^13^C-amide I′ band shifted to higher wavenumbers with increasing admixture of ^12^C-peptide due to the presence of ^12^C-amides in the vicinity of ^13^C-amides. The results indicate that Aβ_40_ and Aβ_42_ form mixed oligomers with a largely random distribution of Aβ_40_ and Aβ_42_ strands in their β-sheets. The structures of the mixed oligomers are intermediate between those of the pure oligomers. There is no indication that one of the peptides forces the backbone structure of its oligomers on the other peptide when they are mixed as monomers. We also demonstrate that isotope-edited infrared spectroscopy can distinguish aggregation modulators that integrate into the backbone structure of their interaction partner from those that do not. As an example for the latter case, the pro-inflammatory calcium binding protein S100A9 is shown not to incorporate into the β-sheets of Aβ_42_.

## Abstract 2

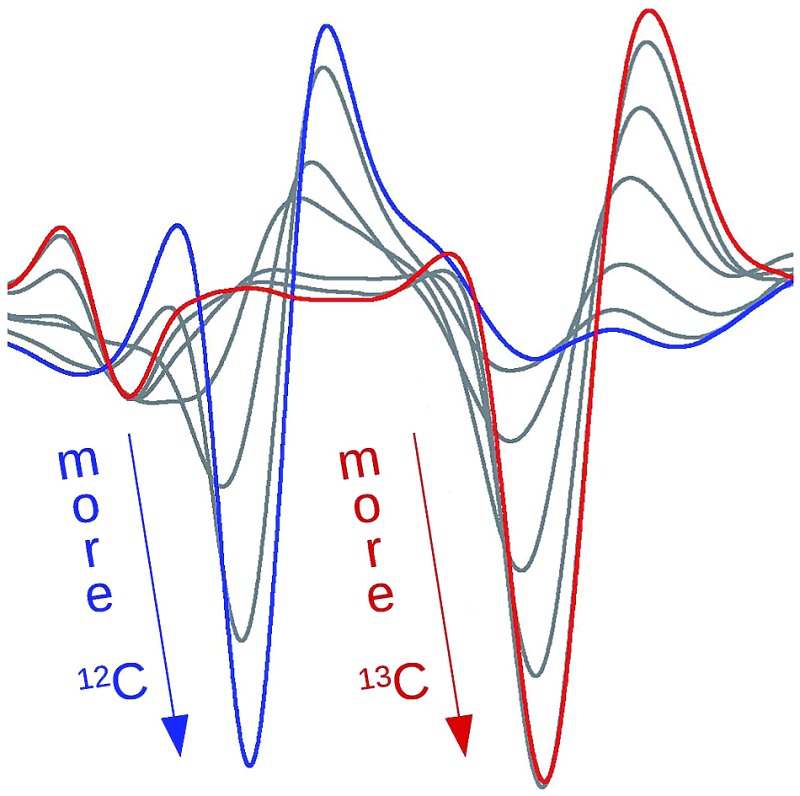
Aβ_40_ and Aβ_42_ co-aggregate and form oligomers with mixed β-sheets as revealed by isotope-edited infrared spectroscopy.

## Introduction

Alzheimer's disease – the most common form of dementia – is associated with the deposition of amyloid fibrils in the human brain. These consist mainly of ∼40 residue-long proteolytic fragments of the β-amyloid precursor protein. The main variants of these amyloid-β (Aβ) peptides are 40 (Aβ_40_) and 42 (Aβ_42_) residues long. Although more of the shorter alloform Aβ_40_ is generated, the longer alloform Aβ_42_ dominates in the Aβ deposits in the human brain.^1^ The Aβ_42_ : Aβ_40_ ratio is enhanced in some familial Alzheimer diseases, which likely causes the early onset of these diseases^2^–^5^ due to increased toxicity from Aβ_42_ 6 and less protection by Aβ_40_.^5^,^7^,^8^ However, the situation is more complex than indicated by this simplistic explanation: a relatively small increase in the Aβ_42_ : Aβ_40_ ratio causes a dramatic increase in toxicity^6^ and mixing two weakly toxic alloforms can generate high toxicity.^9^ Thus, the properties of Aβ mixtures, including their toxicity, cannot simply be extrapolated from the properties of the pure peptides^6^,^9^,^10^ and a recent review concludes that studies of Aβ mixtures “will be crucial in understanding the toxic effects of Aβ”.^11^ One of the most fundamental aspects in this context is whether or not different Aβ alloforms co-aggregate and form common structures. In a wider perspective, cross-reactivity of Aβ with other amyloidogenic proteins^12^–^14^ may link neurodegenerate diseases that are so far considered to be different.

Co-incubation of Aβ_40_ and Aβ_42_ or cross-seeding leads to co-aggregation (cross-reactivity) in early aggregates,^15^,^16^ modifies aggregation kinetics,^6^,^7^,^10^,^17^–^24^ aggregate type distribution,^6^,^10^,^22^,^23^ and may enable the formation of mixed fibrils,^22^,^25^,^26^ although the latter has been disputed recently.^16^ Most of the current evidence for co-aggregation in Aβ oligomers is indirect because either larger aggregates,^10^,^16^–^19^,^22^ or monomeric peptides^7^,^10^ have been observed. Direct evidence for mixing in Aβ oligomers has been obtained,^15^ but using bulky labels attached to Aβ, which may affect aggregation properties. To the best of our knowledge there has not been a study that directly investigates the extent of mixture of Aβ_40_ and Aβ_42_ in Aβ oligomers with chemically unmodified peptides and that investigates the molecular architecture of mixed aggregates. This is in spite of the suggested importance of oligomers^27^,^28^ and of the Aβ_42_ : Aβ_40_ ratio^2^,^6^ for the severity of Alzheimer's disease.

To reveal whether and to what extent Aβ_40_ and Aβ_42_ form mixed aggregates, we used here isotope-edited infrared (IR) spectroscopy. The main present use of this method is to determine the local conformation of polypeptides.^29^–^31^ It has also been applied to separate the spectral contributions of different components in a complex^32^,^33^ but only rarely to detect mixing of two polypeptides^34^,^35^ or of two protein domains^36^ in a common secondary structure. Our work uses the latter approach, provides a computational simulation of the effect, introduces the methodology to determine the randomness of mixing and discusses the sensitivity of the method for this property. We focus on the amide I vibration of the peptide backbone, which is sensitive to secondary structure and to further structural properties like the extension of β-sheets, their twist, the strength of hydrogen bonding, and the relative arrangement of adjacent strands.^37^–^41^ Our results directly demonstrate co-aggregation of Aβ_40_ and Aβ_42_ in oligomeric aggregates. These aggregates are well structured and seem to contain a large proportion of antiparallel β-sheets. Advantageously, the results were obtained with chemically unmodified peptides, without addition of reporter compounds, and in aqueous solution.

## Results and discussion

### Aβ hetero-oligomers with mixed isotope composition

Monomeric, recombinant peptides prepared at alkaline pH in ^2^H_2_O were brought to p^2^H 7.4 and their IR spectra measured against a buffer spectrum after 20 min of incubation as described in ESI.† The preparation led to small-sized oligomers and additional larger aggregates for Aβ_42_ and Aβ_42_-rich mixtures with Aβ_40_ when analyzed by gel electrophoresis after photo-induced crosslinking (see Fig. S5 of ESI†).

**Fig. 1:**
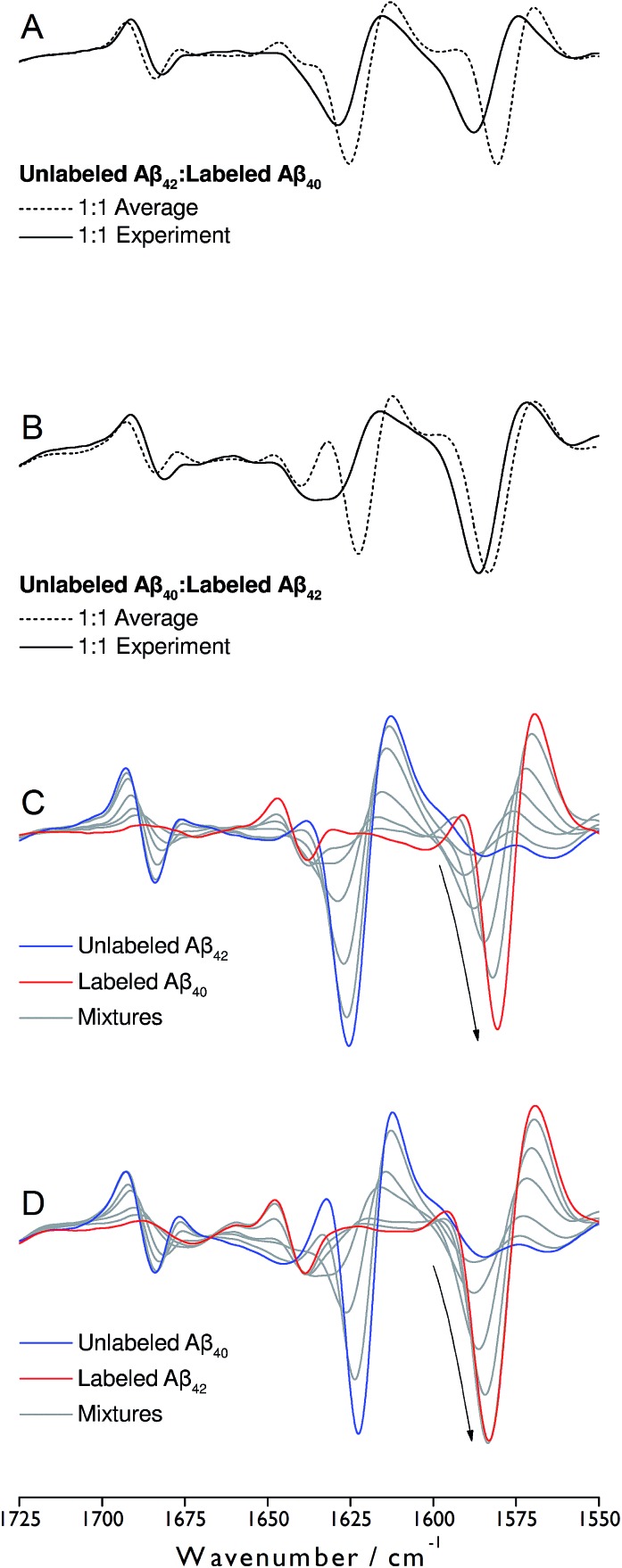
Spectra of the second derivative of IR absorbance of oligomers obtained from mixtures of unlabeled Aβ_42_ and labeled Aβ_40_ (A and C) and unlabeled Aβ_40_ and labeled Aβ_42_ (B and D). Real spectra of 1 : 1 mixtures are shown in panels (A) and (B) (solid lines), together with averaged spectra of the pure compounds (dashed lines). In panels (C) and (D), the spectra of several ratios of unlabeled and labeled peptides (9 : 1, 3 : 1, 1 : 1, 1 : 3, 1 : 9) are shown together with those of the pure oligomers obtained from unlabeled (blue) and labeled (red) peptides. The arrows in panels (C) and (D) indicate the changes in intensity and band position of the main ^13^C-band upon ^13^C-enrichment.

The IR absorbance and second derivative spectra of unlabeled and ^13^C,^15^N-labeled Aβ_40_ and Aβ_42_ show the typical features of oligomers that are commonly assigned to antiparallel β-sheets (see Fig. S1 to S3 and discussion in ESI†). To test whether Aβ_40_ and Aβ_42_ become incorporated into the same oligomers, unlabeled Aβ_42_ was mixed with an equimolar concentration of uniformly ^13^C,^15^N-labeled Aβ_40_ at alkaline p^2^H and incubated at p^2^H 7.4. In a further experiment, labeled and unlabeled peptides were exchanged. The second derivative spectra of the 1 : 1 mixtures are shown in Fig. 1A and B (solid lines). Negative bands in these spectra correspond to component bands in the absorbance spectra. Also shown in Fig. 1A and B are calculated spectra (dashed lines) obtained by averaging the individual oligomer spectra shown in Fig. S1 and S2† (for the corresponding absorbance spectra see Fig. S3†). The calculated spectra are expected when unlabeled and labeled peptides do not mix, but rather give rise to distinct all-unlabeled and all-labeled oligomers. The positions of the main bands in these spectra are the same as in the individual oligomer spectra. In contrast, the real mixture spectra exhibit shifts of all main bands and a loss in intensity of the ^12^C-band (near 1625 cm^–1^). This clearly indicates that the backbones of Aβ_40_ and Aβ_42_ interact in the oligomers and that this interaction influences the vibrational coupling that determines the band position of the β-sheet band.

### Band shifts upon isotopic dilution

To discuss further the structural basis of the Aβ_42_:Aβ_40_ mixing effect, panels C and D of Fig. 1 show spectra recorded for a number of different Aβ_42_ : Aβ_40_ ratios, where one of the peptides was labeled. They reveal gradual upshifts of the main ^12^C- and ^13^C-β-sheet bands (near 1625 and 1580 cm^–1^, respectively) and a downshift of the minor β-sheet band (near 1680 cm^–1^) upon isotopic dilution. Such band shifts upon isotopic dilution have been observed previously.^34^,^36^,^42^–^46^

**Fig. 2:**
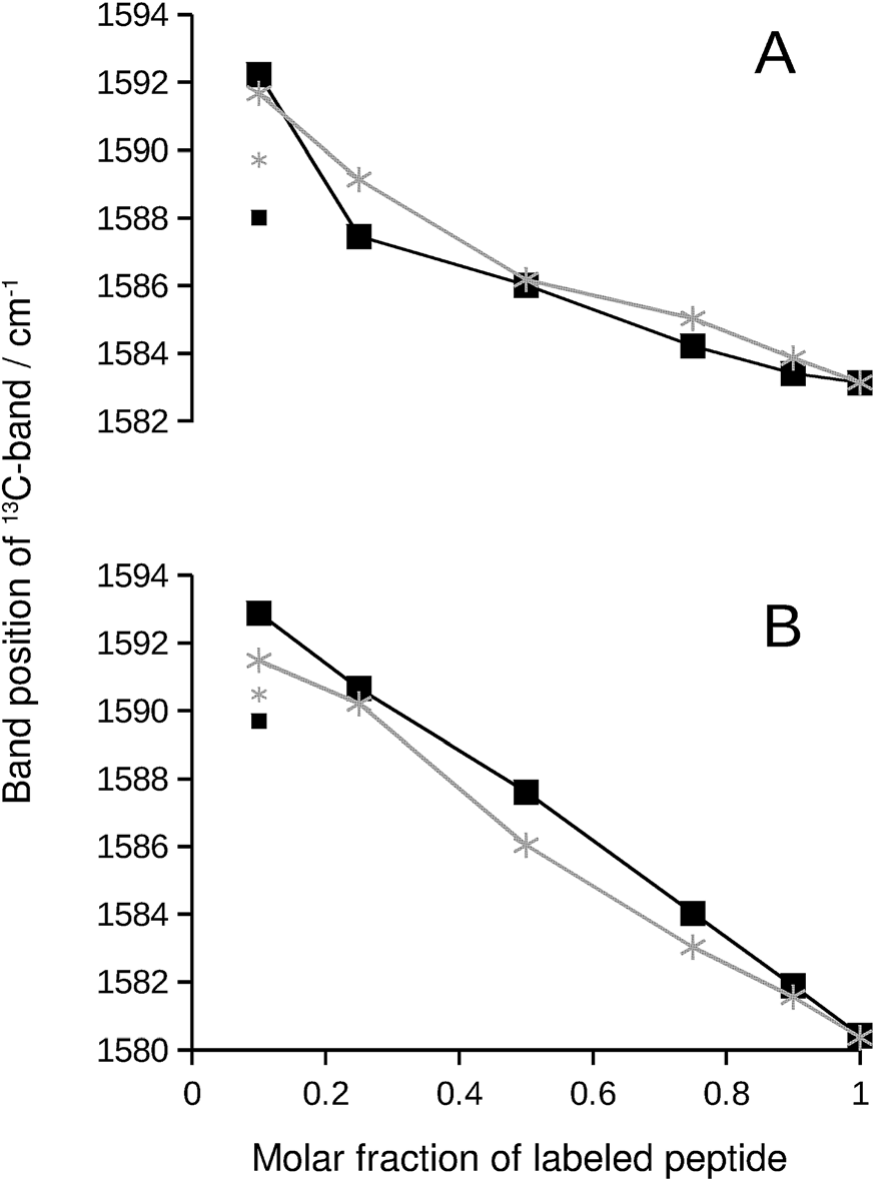
^13^C-band position as a function of the molar fraction of labeled peptide. The black squares and lines are for Aβ_42_:Aβ_40_ mixtures, where one of them was labeled. The gray stars and lines are for mixtures of labeled and unlabeled versions of the same peptide. (A) ^13^C-Aβ_42_:^12^C-Aβ_40_ (black) and ^12^C-Aβ_42_:^13^C-Aβ_42_ (gray) mixtures. (B) ^12^C-Aβ_42_:^13^C-Aβ_40_ (black) and ^12^C-Aβ_40_:^13^C-Aβ_40_ (gray) mixtures. The large symbols indicate band positions that were directly determined from the second derivative spectra except for the data points at 0.1 molar fraction of labeled peptide, which were obtained from a fit as described in ESI.† The small symbols show the original band positions in the second derivative spectra for these data points. Note that the data for completely labeled peptides in each panel are from two independent series of experiments. The agreement between the band positions demonstrates the excellent reproducibility of the spectra.

We chose the ^13^C-band for further evaluation, because it can be detected even at low ^13^C : ^12^C-ratio in second derivative spectra. The position of this band is plotted in Fig. 2 and reflects the continuous downshift of the band upon ^13^C-enrichment. The different end points of the curves (when only labeled peptide is present) reflect the different structures of the pure oligomers (see Fig. S2†).

We explain the observed ^13^C-band shift by the formation of β-sheets with mixed isotopic composition, *i.e.* sheets that consist of ^12^C- and ^13^C-strands. For the experiments shown in Fig. 1C and D, this means that the sheets contain strands from Aβ_40_ and Aβ_42_. This interpretation will be tested in the following sections by calculations and control experiments.

### Calculated amide I spectra of antiparallel β-sheets with mixed isotope composition

To support our explanation for the shift of the ^13^C-band upon isotopic dilution we discuss now spectrum calculations that simulate the above experiments. The aim was to reproduce the effects of isotopic dilution in a qualitative way.

Details of the calculations are provided in ESI.† In brief, amide I spectra were calculated using coupling constants from density functional theory for nearest neighbor interactions and from transition dipole coupling for other interactions. Diagonal and non-diagonal elements of the mass-normalized force constant matrix considered the carbon isotope of the respective amide group(s). Spectra were calculated from 3000 sheets with a statistical distribution of ^12^C- and ^13^C-strands at a given ^13^C : ^12^C ratio. Errors in calculated band positions were obtained by repeating the calculations 20-times.

**Fig. 3:**
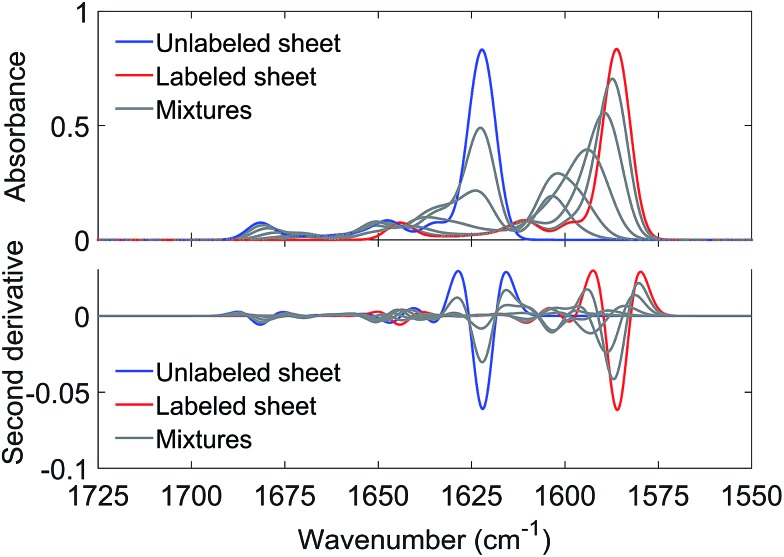
Calculated amide I spectra for an antiparallel β-sheet with 6 strands and 10 residues per strand (9 complete amide groups). The blue and red spectra were calculated for entirely unlabeled and labeled sheets, respectively. The gray spectra were calculated for different molar fractions of ^13^C-peptides, as used in the experiments (0.1, 0.25, 0.5, 0.75, 0.9). Each simulated spectrum is the average of the spectra from 60 000 sheets with a statistical distribution of labeled and unlabeled strands at a given ^13^C : ^12^C-ratio. ^13^C-enrichment gradually shifts the ^13^C-band from 1603.5 to 1586.2 cm^–1^. The ^12^C band position for the completely unlabeled sheet is 1622.2 cm^–1^.

Antiparallel β-sheets of different sizes generated qualitatively similar spectra. As an example, Fig. 3 shows results obtained with a sheet of 6 strands and 10 residues (9 complete amide groups) per strand. The main ^12^C-band near 1622 cm^–1^ loses intensity already at low ^13^C : ^12^C-ratios whereas the ^13^C-band is observed for all ratios. It shifts gradually down with increasing ^13^C : ^12^C-ratios as observed in the experiments. These simulations show that the experimental result can be explained by mixing strands with different carbon isotopes in the same β-sheet. This conclusion is independent from the molecular architecture of the β-sheets as qualitatively similar results were obtained for parallel sheets (see Fig. S7†).

While the band positions of the completely labeled and unlabeled 6-stranded sheet are in reasonable agreement with the experimental values, the ^13^C-band shift between 10% and 100% ^13^C content is calculated to be larger than experimentally observed (17 cm^–1^*versus* 9–12 cm^–1^ observed in the four mixing experiments shown in Fig. 2). The calculated shift is smaller for a parallel β-sheet of the same size (14 cm^–1^) but these calculations fail to reproduce the high wavenumber band that is clearly observed in the experimental spectra (compare Fig. 1 and S7†). The discrepancy is not due to our use of ideal β-sheet structures for the calculations, as similar or larger shifts were obtained for a 6-stranded antiparallel sheet from a streptavidin mutant (20 cm^–1^) and in a calculation for an ideal antiparallel sheet in which the vibrational coupling constants were varied statistically to simulate structural disorder (17 cm^–1^).

The likely reason for the discrepancy between the experimental and the calculated ^13^C-band shift is the internal structure of the oligomers as the shift is sensitive to the number of adjacent strands that contain the same carbon isotope.^36^,^46^ When this number is one, the calculated ^13^C-band shift (between 10% and 100% ^13^C content) was 24 cm^–1^ for large parallel sheets.^36^,^46^ When this number is two, the shift decreased to 18 cm^–1^. The shift decreased further when the number of adjacent strands with the same carbon isotope was increased to three or four. We confirmed this trend in our own calculations with 6-stranded sheets that were composed of building blocks of two adjacent β-strands with the same carbon isotope. The shift was now reduced to 10 cm^–1^, which compares favorably to the 9–12 cm^–1^ shift in the four mixing experiments shown in Fig. 2. Therefore a plausible explanation of our results is that each peptide contributes two or more adjacent strands to the oligomers. Our shifts are smaller than those calculated by Moran *et al.* because we use sheets with a much smaller number of strands.

### Explanation of the band shifts upon isotopic dilution

**Fig. 4:**
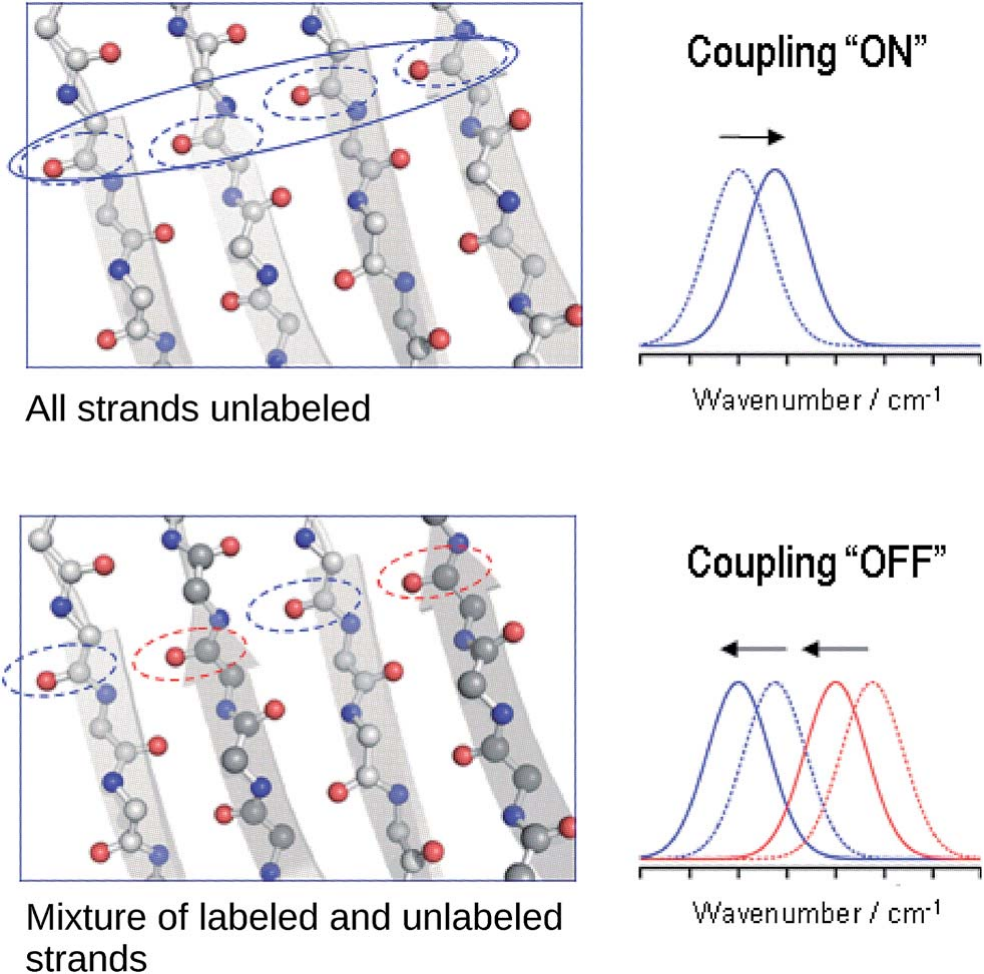
Simplified explanation for the spectral effects of mixing labeled and unlabeled strands in a β-sheet. In the sheet, carbon, nitrogen and oxygen atoms are shown in gray, blue and red, respectively. The dashed ellipses denote individual amide I oscillators, they are blue for ^12^C-amides and red for ^13^C-amides. The solid ellipse denotes a delocalized vibration due to coupling of the indicated individual oscillators. The dashed and solid spectra on the side show the main β-sheet absorption band before and after coupling is established (top), as well as before and after it is partially broken (bottom) for the main ^12^C- (blue) and the main ^13^C-band (red).

The reason for the spectral shifts can be found in the decreased coupling between neighboring oscillators in a β-sheet with mixed isotope composition compared to an all-unlabeled or all-labeled sheet. This is shown schematically in Fig. 4 using an ideal anti-parallel β-sheet to illustrate the principle. In a β-sheet composed entirely of either unlabeled or labeled strands, such as the one shown in the top panel, the local amide I′ oscillations of individual peptide groups (dashed ellipses) couple strongly because (1) they have a similar vibrational frequency, (2) the main component of their transition dipole moments is oriented in the same direction and (3) they are close in space. This delocalizes the vibrations over up to 12 strands^47^ (solid ellipse). The strongest coupling of a particular amide group in an antiparallel β-sheet is with the two hydrogen bonded amide groups in the adjacent strands and with the diagonally opposed amide group in one of the adjacent strands.^37^,^48^ Intrastrand coupling between nearest neighbors is smaller but still significant. Nevertheless and to simplify the illustration, only the strongest interaction is considered in Fig. 4. Coupling affects band positions and leads to a downshift in case of the main β-sheet band. This is shown in the set of spectra on the right side of the top panel of Fig. 4. Stronger coupling leads to a larger downshift, which increases with sheet flatness and number of incorporated strands (usually up to 10 strands)^37^–^40^ and is, among others, the reason for the downshifted absorption of β-sheets in amorphous aggregates compared to those in soluble proteins. The above discussion indicates that IR spectroscopy is sensitive to structural variations in β-sheets that occur within a length scale of 10–12 strands.

In the case of an isotopically mixed β-sheet (bottom panel of Fig. 4), where fully unlabeled and fully labeled strands alternate randomly, the difference in the intrinsic vibrational frequencies of the amide oscillators makes that unlabeled (blue ellipses) and labeled (red ellipses) oscillators couple only weakly. Therefore, the vibration is less delocalized over the span of the sheet and more localized on the individual strands. The net effect is that the vibrational frequencies of both unlabeled and labeled oscillators are closer to those of the uncoupled, hydrogen-bonded amide carbonyls (∼1645 cm^–1^ for ^12^C=O, and ∼1600 cm^–1^ for ^13^C=O), and the corresponding β-sheet absorption bands shift to higher wavenumbers compared to a fully unlabeled or fully labeled sheet.

The opposite phenomenon occurs for the high wavenumber band of anti-parallel β-sheets, which experiences a downshift upon loss of coupling. The smaller magnitude of the shift, together with the moderate intensity of this band, makes it less useful in the study of amyloid-β oligomers compared to the main β-sheet band.

### Control: Aβ homo-oligomers with mixed isotope composition

If the ^13^C-band shift indicates isotopically mixed β-sheets, then similar shifts must be observed when labeled and unlabeled versions of the same peptide are mixed in homo-oligomers. In such experiments, labeled and unlabeled peptides form randomly mixed β-sheets because they are chemically identical. Our experiments with homo-oligomers of varying isotope composition resulted in similar ^13^C-band shifts, which are included as gray lines in Fig. 2. The results with homo-oligomers support our interpretation that Aβ_42_:Aβ_40_ hetero-oligomers form mixed β-sheets.

### Evidence for random mixing in hetero-oligomers

The ^13^C-band shifts of the homo-oligomers shown in Fig. 2 serve as reference curves for random mixing. They superimpose well with the curves for Aβ_42_:Aβ_40_ mixtures indicating that these two peptides form common β-sheets which contain a random or close to random mixture of Aβ_40_ and Aβ_42_ strands.

In our experiments, Aβ_42_:Aβ_40_ oligomers of different sizes are formed (see ESI†), which might have different mixing preferences. The limiting cases are randomly mixed Aβ_42_:Aβ_40_ oligomers on the one hand and chemically homogeneous aggregates, *i.e.* pure Aβ_42_ or pure Aβ_40_ oligomers, on the other hand. Intermediate cases are also conceivable. When one of the two Aβ alloforms is labeled, such different kinds of oligomers will have different ^13^C-band positions for a given isotope ratio according to the calculations shown in Fig. S8.† The ^13^C-band position will be lower for the aggregates with higher purity than for aggregates with a statistical Aβ_42_:Aβ_40_ composition. Experimentally, these different band positions will be difficult to distinguish because the bands are likely to overlap. The overlap will lead to a single broad ^13^C-band that is downshifted with respect to that for randomly mixed aggregates at all isotope ratios. This is not observed. Instead, ^13^C-band positions for the heterogeneous Aβ_42_:Aβ_40_ mixtures (black curves in Fig. 2) superimpose well on those for homogeneous oligomers, which have a statistical distribution of labeled and unlabeled strands (gray curves in Fig. 2). Therefore none of the dominant oligomers in our Aβ_42_:Aβ_40_ samples deviates enough from the random mixing case to produce a detectable effect.

### Control: Aβ hetero-oligomers from unlabeled peptides

**Fig. 5:**
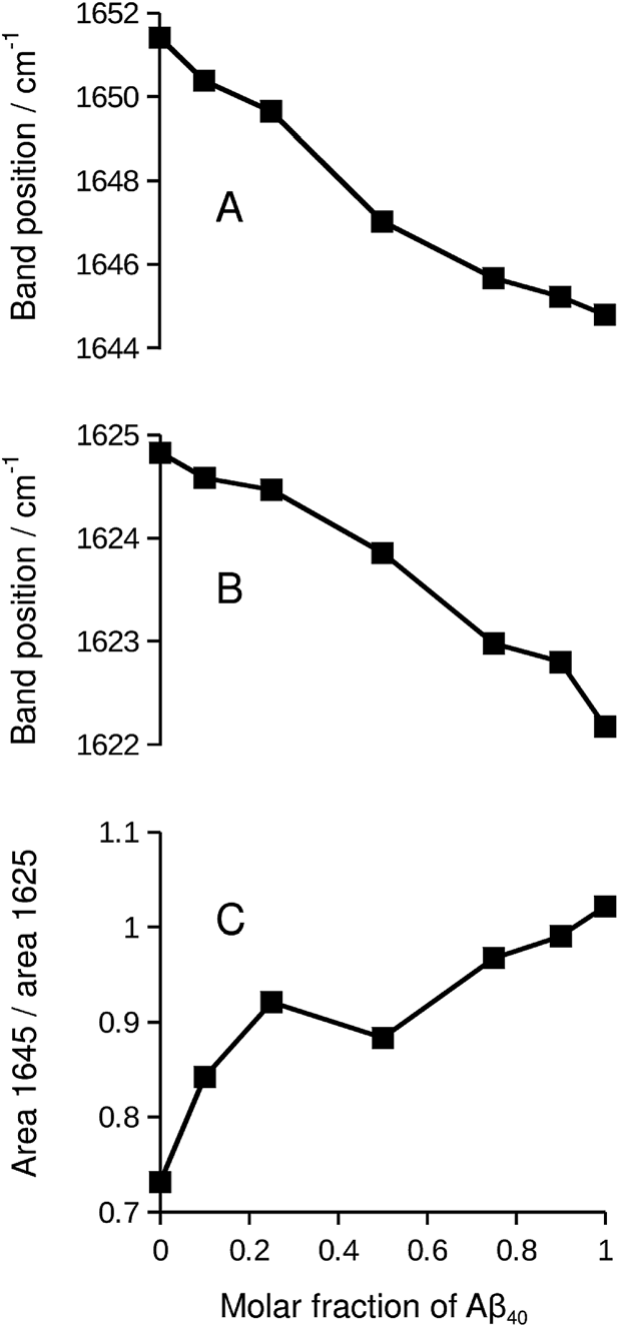
Spectral changes upon increasing the Aβ_40_ content in Aβ_42_:Aβ_40_ mixtures of unlabeled peptides. (A) Band position of the broad band near 1650 cm^–1^ in the second derivative spectra. Only for this panel, the smoothing range for calculating the second derivative was increased from our standard value of 13 points for determining band positions to 25 points to obtain a more accurate description of the small band of the Aβ_42_ sample. (B) Band position of the β-sheet band near 1625 cm^–1^ in the second derivative spectra. (C) Integrated absorbance near 1645 cm^–1^ divided by the integrated absorbance near 1625 cm^–1^. Integration in the ranges 1650–1640 cm^–1^ and 1630–1620 cm^–1^ was done with method E of the Bruker OPUS software with respect to a baseline drawn between the averaged absorbance in the 1700–1695 cm^–1^ and in the 1610–1605 cm^–1^ range.

While the isotope effect evident in Fig. 1 and 2 reveals mixing of Aβ_40_ and Aβ_42_, it masks band shifts due to structural differences between the homo-oligomers and the hetero-oligomers. Therefore we repeated the experiment with unlabeled peptides. When unlabeled Aβ_40_ and Aβ_42_ were mixed, both the band position of the main β-sheet and that of the broad band near 1645 cm^–1^ shifted in a nearly linear fashion between the extreme values of the pure compounds. This is shown in panels A and B of Fig. 5. Please note that this series of experiments is completely independent from the ones shown in Fig. 1. Therefore the band positions of the pure compounds are slightly different. The band position of the Aβ_42_ spectrum in Fig. 5 (1624.8 cm^–1^) is the lowest measured for six independent samples (average 1625.5 ± 0.4 cm^–1^). The band position for Aβ_40_ (1622.2 cm^–1^) in Fig. 5 is very close to the average of 1622.3 ± 0.1 cm^–1^ determined for three samples.

Concomitant with the shifting band positions, the broad band near 1645 cm^–1^ intensifies as the Aβ_40_ content increases (see also Fig. S1†). This may indicate that there is more random coil structure in the Aβ_40_ oligomers. However, intensities in second derivative should be compared with care as they also depend on the band width. Therefore we sought confirmation for the above interpretation in the respective absorbance spectra and found it confirmed: the absorbance spectrum of Aβ_40_, but not that of Aβ_42_, shows a pronounced shoulder near 1640 cm^–1^ that can be assigned to random coil structures as shown in Fig. S3.† In the mixtures, the absorbance in the 1650–1640 cm^–1^ region increases relative to that in the 1630–1620 cm^–1^ region as the Aβ_40_ content increases (panel C of Fig. 5).

As the curves in Fig. 5 are nearly linear, they indicate a gradual transition between the spectra of the pure oligomers and thus a gradual shift between the backbone structures of Aβ_42_ oligomers and of Aβ_40_ oligomers as the peptide composition of the oligomers changes. This result has several implications:

(i) As a control experiment it indicates that the backbone structures of the mixed aggregates are similar to those of the pure oligomers. The shift of the main β-sheet band (panel B) is much smaller than the ^13^C-band shift in Fig. 5. Therefore, a conformational effect can be excluded as an explanation for the ^13^C-band shift. Note also that the shifts in Fig. 5 depend on the Aβ_42_ : Aβ_40_ ratio, whereas those in Fig. 1 and 2 depend on the ^13^C : ^12^C-ratio. In the latter experiments, increasing the Aβ_40_ content leads to a downshift of the ^13^C-band when Aβ_40_ is labeled, but to an upshift when it is not labeled.

(ii) From a methodological perspective, Fig. 5 demonstrates that mixing can only be detected with the help of isotope labels (Fig. 1) because the curves in Fig. 5 are close to those expected for the case when Aβ_40_ and Aβ_42_ do not mix.

(iii) Regarding the biological system, Fig. 5 shows that none of the peptides forces the backbone structures of its pure oligomers on the mixed aggregates when the two peptides co-aggregate from a mixture of monomers. Otherwise, the curves in Fig. 5 should be more curved.

### Control: Aβ_42_ and S100A9

The ability of Aβ_42_ and Aβ_40_ to form mixed oligomers has the natural consequence that they mutually influence their aggregation properties. In contrast, other interaction partners may affect aggregation without integrating into the backbone structure of the Aβ aggregates. In the following we will show that isotope-edited IR spectroscopy can distinguish between these two cases and thus provides information that is complementary to that obtained from other aggregation assays.

As an example for the second case, we chose the brain-expressed, pro-inflammatory calcium binding protein S100A9, which is also called migration inhibitory factor-related protein 14 (MRP14) or calgranulin B. It forms amyloids on its own and is known to interact with Aβ, which enhances the amyloidogenicity of both.^49^,^50^ In this case the interaction proceeds *via* transient hydrophobic contacts involving side chains,^49^,^50^ rather than *via* direct interaction between backbone segments of the two proteins.

**Fig. 6:**
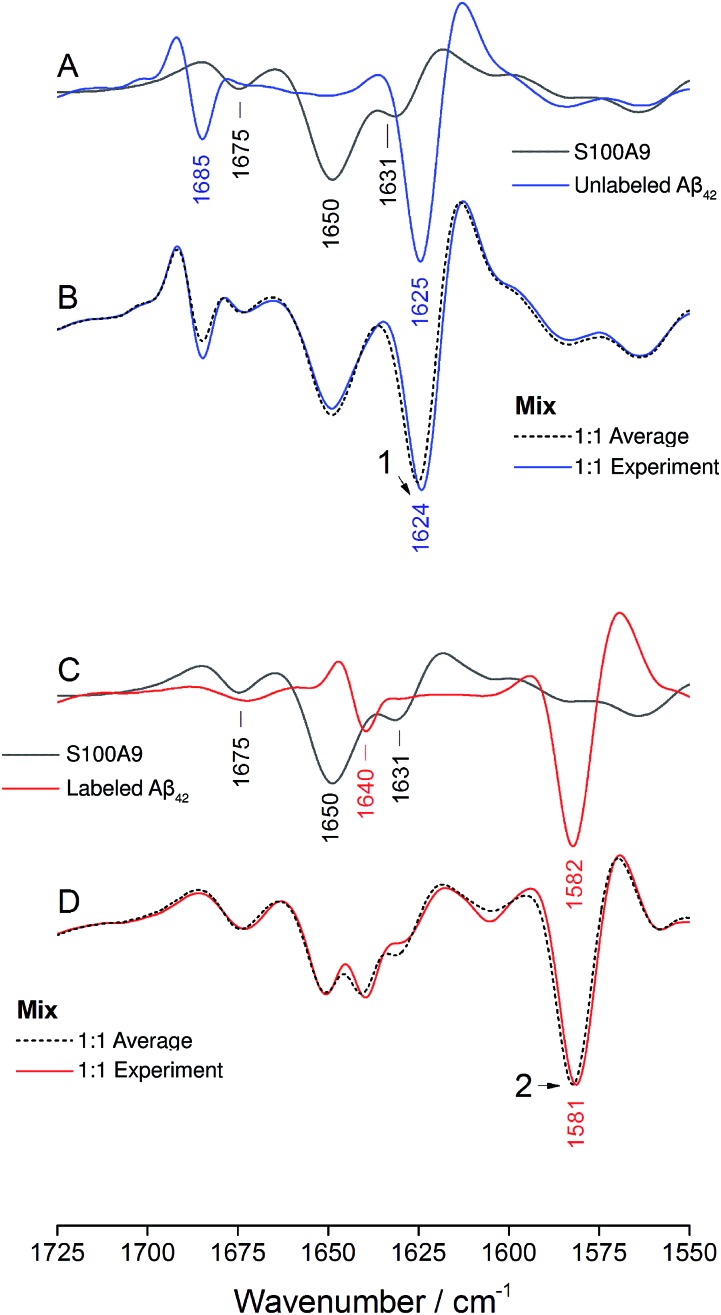
Oligomer formation by unlabeled and labeled Aβ_42_ in the presence of S100A9. Panels (A), (B) and (C), (D) refer to experiments performed using unlabeled and labeled Aβ_42_, respectively. S100A9 is unlabeled in both experiments. Panels (A) and (C) show spectra of the pure components and panels (B) and (D) spectra of the 1 : 1 mixtures (w/w). “Experiment” refers to the experimental spectrum and “Average” to the average spectrum of the pure components, which is the expected mixture spectrum in the absence of structural changes.

The IR absorption spectrum of S100A9 in the amide I′ region is shown in black in panels A and C of Fig. 6. It exhibits three main components. A strong, sharp band at 1650 cm^–1^ originates from α-helices, the dominant secondary structure in the protein. The minor band at 1631 cm^–1^, usually assigned to β-sheet structures, rather originates from solvent-exposed and highly hydrated stretches of α-helices as there are no β-sheets in this protein.^51^ The band at 1675 cm^–1^ originates from turns.

The spectrum of the mixture with unlabeled Aβ_42_ oligomers is shown as blue spectrum in panel B together with a calculated spectrum of the 1 : 1 mixture (average spectrum of the pure components, dashed spectrum). The two spectra are virtually superimposable, with all the main features of the Aβ_42_ oligomer spectrum being preserved. A small (∼1 cm^–1^) downshift of the main β-sheet band can be observed (arrowhead 1) and is likely due to accelerated aggregation in the presence of S100A9. This interpretation is based on the observation that the position of this band downshifts with increased aggregation time.^52^

The same experiment was performed with labeled Aβ_42_ oligomers, as shown in panels C and D. Again, the calculated (dashed) and experimental (solid red) spectra of the 1 : 1 mixture are virtually superimposable and a small downshift of the main β-sheet band of labeled Aβ_42_ oligomers can be observed (arrowhead 2). In contrast to the Aβ_40_ and Aβ_42_ mixtures, no upshift of the main ^13^C-β-sheet band occurs when S100A9 is added to Aβ_42_. This indicates that the upshift observed for Aβ_40_:Aβ_42_ mixtures is not induced by transient, hydrophobic interactions between side chains as they occur between S100A9 and Aβ.

## Conclusions

Our experiments and control experiments clearly demonstrate that Aβ_40_ and Aβ_42_ form mixed oligomers, when they are mixed as monomers before aggregation is initiated. Mixing occurs on the level of secondary structure, where the Aβ_40_ backbone is in direct interaction with the Aβ_42_ backbone in the β-sheets of the oligomers. Mixing of Aβ_40_ and Aβ_42_ strands in the β-sheets is random or close to random. This structural information was inferred from band shifts in the IR spectrum, when one of the peptides was ^13^C-labeled. The different carbon isotopes in the mixed sheets disrupt the interstrand vibrational coupling, which leads to an upshift of the main β-sheet band. This conclusion is also supported by our spectrum calculations, which reproduce the shifts observed in the experiments. The best agreement between calculations and experiments is obtained when we assume that each peptide contributes two adjacent strands to the oligomers.

The backbone structures of mixed Aβ_42_:Aβ_40_ oligomers are intermediate between those of the pure aggregates. There is no indication that one of the alloforms forces its preferred structure on the other alloform when they are mixed as monomers. This conclusion is based on band shifts observed for mixtures of unlabeled peptides, which depend linearly on the composition of the aggregates.

The random or close to random mixing of Aβ_40_ and Aβ_42_ in the β-sheets of oligomers occurs in spite of their different propensities to aggregate^6^,^10^,^16^,^21^,^23^ and the different oligomer structures that they adopt^23^ (see also Fig. S1†). Our findings are in line with previous more qualitative conclusions using chemically modified peptides^15^ or peptides attached to a support^10^ and with conclusions drawn indirectly from the observation of fibril formation.^16^ Our work adds information on the extent of mixing and on the structural architecture of mixed oligomers. It strengthens the view that heterogeneous oligomers are relevant for Alzheimer's disease. In addition, it highlights the necessity to consider them in the amyloid field in general, since similar mixed oligomers may assemble under many other amyloidogenic conditions in several diseases.

## Conflicts of interest

There are no conflicts to declare.

## Supplementary Material

Supplementary information
