# Supplementary material for: Amyloid β-peptides 1–40 and 1–42 form oligomers with mixed β-sheets
Source: Chem Sci. 2017 Oct 12;8(12):8247–54. doi: 10.1039/c7sc01743j (PMC5857929; doi:10.1039/c7sc01743j)
Supplement: Supplementary file 1 [file SC-008-C7SC01743J-s001.pdf]

Electronic Supplementary Material (ESI) for Chemical Science.
This journal is © The Royal Society of Chemistry 2017

Electronic Supporting Information

Amyloid β-peptides 1-40 and 1-42 form oligomers with mixed β-sheets

Maurizio Baldassarre, Cesare M. Baronio, Ludmilla A. Morozova-Roche, and Andreas Barth

Materials and Methods

Materials

Recombinant (trifluoroacetic acid-free) $A\beta_{40}$ (MW 4329.9 Da) and $A\beta_{42}$ (MW 4514.1 Da) with ammonium acetate as a counter ion were purchased from AlexoTech (Umeå, Sweden). Recombinant, uniformly ^{13}C,^{15}N-labeled $A\beta_{40}$ (MW 4576.9 Da) and $A\beta_{42}$ (MW 4772.1 Da) were obtained from the same producer as above and had approx. 90% isotope incorporation. The purity of the peptides was 95% according to the manufacturer. Deuterium oxide was from Cambridge Isotope Laboratories (Tewksbury, Massachusetts, USA). S100A9 was prepared as described previously.[1]

Solubilization of lyophilized Aβ samples

Lyophylized Aβ samples were resuspended in ice-cold HFiP at a concentration of 1 mg/mL. The solution was kept on ice for 30 min, sonicated in an ice-water mixture for 5 min and eventually inspected for optical clarity. If no visible particles could be observed, the solution was let to equilibrate at room temperature for 30 min and then aliquoted in low-binding Eppendorf tubes, 50 µL/tube. The tubes were kept in a beaker with their lids open and the beaker was covered with a laboratory paper wipe to avoid deposition of dust particles. The beaker was then kept in a fume hood overnight to allow for HFiP evaporation. Trace HFiP was removed by keeping the tubes in a SpeedVac evaporator for 2 h under strong vacuum. This procedure yielded a clear peptide film on the bottom of each tube. The films were overcast with N_2 gas, sealed with parafilm and stored at –20°C until use.

Resolubilization and mixing of monomeric Aβ peptides

Monomeric Aβ of the various types was obtained by resuspension of the HFiP-treated films in 20 mM ice-cold NaO^2H. The solution was gently pipetted up and down the tube walls to achieve a complete resuspension of the film. Care was taken not to introduce air bubbles. Depending on the exact molecular weight of the Aβ species, the volume of NaO^2H used ranged between 52 and 55 µL, which gave in all cases a final peptide concentration of 200 µM. The solubilization was allowed to proceed for at least 1 h, during which the peptide solution was constantly kept on ice. This procedure yielded monomeric and disaggregated Aβ peptides, as judged by the typical random coil signals observed in both infrared (IR) and circular dichroism (CD) spectra.

All procedures involving resuspension, handling and mixing of peptides in aqueous solutions were carried out in a custom-made glove box continuously purged with dry N_2 gas. This avoided contamination of samples prepared in ^2H_2O with atmospheric moisture and CO_2.

The monomeric Aβ peptide solutions in NaO^2H were mixed in low-binding tubes to yield various peptide ratios in a volume of 10 µL. The mixtures were mixed by gentle pipetting and kept on ice until analysis.

In situ induction of oligomer formation

Oligomer formation by monomeric Aβ solutions or mixtures in NaO^2H was induced by rapidly changing the p^2H to a value of approx. 7.4, from an initial value that was typically >11. This was achieved by drying 4 µL of 100 mM sodium phosphate, pH 7.0, under vacuum on a UV-grade CaF_2 window. The film was redissolved in 10 µL ^2H_2O and dried again. This procedure was repeated twice. Eventually, the dried buffer film was resuspended using 4 µL of the various peptide solutions. A 50-µm plastic spacer, covered on both sides with a small amount of vacuum grease, was laid on the CaF_2 window and a flat CaF_2 window was added. A second sample was prepared in parallel in the same way, except that 20 mM NaO^2H was used to rehydrate the dried buffer film. This sample served as a blank for the spectroscopic analysis. All procedures were carried out in a glove box in a N_2 atmosphere.

Oligomer formation in the presence of S100A9

Lyophilized S100A9 was dissolved in 100 mM sodium phosphate, p^2H 7, at a concentration of 1.8 mg mL^{-1}. The protein solution was incubated overnight at 4 °C to allow for $^1H/^2H$ exchange. Unlabeled and labeled $A\beta_{42}$ films were resuspended in 20 mM NaO^2H at a concentration of approx. 1.8 mg mL^{-1}, and incubated on ice for at least 1 h. To induce oligomer formation, 2 µL of the S100A9 solution was mixed with an equal volume of peptide solution directly on a CaF_2 window. This procedure decreases the concentration of each species to 0.9 mg mL^{-1}, and changes the p^2H to approx. 7.4, which induces oligomer formation by the peptide. To obtain IR spectra of S100A9 and unlabeled/labeled $A\beta_{42}$ alone, the former was mixed to 20 mM NaO^2H, while the latter was mixed with 100 mM sodium phosphate, p^2H 7.

IR spectroscopy

The peptide sample and the corresponding blank were mounted on a two-position sample shuttle in a Tensor 37 Fourier transform IR spectrometer (Bruker, Germany) equipped with an MCT detector cooled with liquid nitrogen. The spectrometer and its sample compartment were continuously purged with CO_2-free, dry air. A waiting time after closing the sample compartment lid permitted removal of moisture and CO_2 introduced during sample loading. The total time between lowering the p^2H to ~7.4 and the measurement was 20 min. The temperature of the sample and buffer cells was constantly kept at 20 °C by means of an external water bath.

Interferograms were recorded at a resolution of 2 cm^{-1}, apodized using a 3-term Blackman–Harris apodization function and Fourier-transformed with a zero-filling factor of 2. Sixteen consecutive interferograms were averaged to obtain a single sample or buffer

spectrum. Each final peptide spectrum was obtained by averaging 32 individual spectra and therefore corresponded to 512 scans.

The use of a sample shuttled allowed for automatic removal of residual water vapor signals. We placed a buffer sample into the background slot of the shuttle, which resulted in the subtraction of most of the ^2H_2O buffer spectrum and enabled the use of larger apertures as compared to an empty background. Larger apertures lead to higher signal-to-noise ratios as demonstrated previously.[7] This resulted in high-quality, water vapor-free spectra of Aβ peptide samples at a concentration of 0.9 mg mL^{-1} (200 µM), which is low for IR spectroscopy.

IR spectra were recorded and analyzed using the OPUS software from the instrument manufacturer. In the measured absorbance spectra, most of the buffer signal was already subtracted due to a buffer sample in the background position of the sample shuttle. For absorbance spectra shown in Fig. S3 and used for evaluating the ^{13}C-band position at 0.1 molar fraction of labeled peptide, residual buffer and solvent contributions were subtracted using an experimental buffer spectrum in ^2H_2O and an *in silico* $^1H^2HO$ spectrum for the $^1H^2HO$ band near 1460 cm^{-1}. The latter was generated by a fit to one of the experimental spectra and consisted of a main band at 1456 cm^{-1} and a minor band at 1477 cm^{-1}.

Second derivative spectra were calculated directly from the measured absorbance spectra using a smoothing range of 17 data points (approx. 17 cm^{-1}) for spectra shown in figures and 13 data points for evaluating band positions. Subtraction of residual buffer and solvent contributions as described above did not change the band positions in second derivative spectra and was not used except for determining the band position of the ^{13}C-band at a molar fraction of labeled peptide of 0.1. This position was affected by overlap of other bands including the main ^{12}C-band near 1625 cm^{-1} and a side chain band near 1585 cm^{-1} that is clearly present in the spectrum of unlabeled peptides. Therefore the band position of the ^{13}C-band was determined by curve fitting after subtracting residual solvent and buffer contributions as described above. The thus obtained absorbance and second derivative spectra were then fitted simultaneously as described previously,[8] using a factor (weight) of 300 to increase the amplitude of the second derivative spectrum. The fitting range was 1730-1550 cm^{-1} and the fit model consisted of 11 bands placed initially at 1730, 1683, 1678, 1664, 1653, 1632, 1624, 1615, 1590, 1586, and 1560 cm^{-1}. The main motivation for the 1586 cm^{-1} band was its presence in the ^{12}C-spectra and including it increased the fit quality slightly. All other bands were motivated by the shape of the second derivative spectrum. For example, the band near 1632 cm^{-1} was needed to decrease the positive side lobe of the band near 1624 cm^{-1} and the band placed initially at 1615 cm^{-1} corresponds to an inflection in the second derivative spectra near 1605 cm^{-1}.

CD spectroscopy

CD spectra of the IR samples were recorded immediately after IR analysis. The IR cell was fitted into a custom-made adapter and inserted in the spectropolarimeter. Spectra were recorded by means of a ChiraScan spectropolarimeter (Applied Photophysics, state). Forty individual spectra were recorded and averaged to give a single peptide spectrum.

Photo-induced cross-linking

Photo-induced cross-linking of unmodified peptides (PICUP) was performed according to the method originally developed by Bitan et al.[9,10] with some modifications. To perform cross-linking on the samples, the windows of the IR cell were opened by tilting one of the windows relative to the other so that the sample droplet could be reached with a thin pipette tip. The peptide droplet (4 µL) was mixed *in situ* with 6 µL of a solution consisting of the following: 4 µL ddH_2O, 1 µL 4 mM tris(bipyridyl)Ru(II) and 1 µL 80 mM ammonium persulfate. The solution was mixed thoroughly and transferred to a clear Real-Time PCR tube. The tube was illuminated for 1 s using an AvaLight deuterium/halogen light source (Avantes, the Netherlands) controlled by a PC. 10 µL tricine sample buffer (Bio-Rad, USA) containing 5% 2-mercaptoethanol were immediately added to avoid further cross-linking induced by ambient light. The tube was frozen in liquid nitrogen and stored at –80°C.

Oligomer size analysis

The molecular weight distribution of the cross-linked Aβ oligomers was determined by SDS-PAGE analysis. To this purpose, the frozen cross-linked oligomers were let to thaw at room temperature and then 20 µL (approx. 3.5 µg peptide) were loaded onto 10–20% tris-tricine pre-cast gels (Bio-Rad). Molecular weight standards (Dual Xtra Standards, Bio-Rad) were also loaded (2 µL markers + 8 µL sample buffer). The gels were mounted onto a Mini-PROTEAN electrophoresis system (Bio-Rad) and run as per manufacturer's instructions. After the electrophoretic run, the gels were stained using a commercially available silver staining kit (Thermo-Fisher Pierce) and imaged using Intelligent Dark Box II (Fujifilm).

Calculation of amide I spectra

Published atomic coordinates for ideal antiparallel[11] and parallel[12] β-sheets were used to generate β-sheet building blocks. β-sheets of a specified even number of strands and a specified even number of residues per strand were then created by copying and translating the building block. We discuss here mainly sheets with 6 strands and 10 residues (9 complete amide groups). For antiparallel sheets, the building block consisted of two residues in one strand and their neighbors in one adjacent strand. For parallel sheets, the building block had 2 residues in one strand. The *z*-coordinate was multiplied with -1 to transform the original coordinates with (*R*)-configuration to (*S*)- configuration.[13] To compare the ideal β-sheets with a real structure, we also used the 6-stranded antiparallel β-sheet of residues 37-44, 53-61, 71-80, 87-96, 103-112, 122-130 from chain B of a streptavidin mutant (pdb code 2Y3E).[14] This sheet had slightly less residues in several strands than the ideal sheet that we consider, but test calculations with an ideal sheet with the same number of residues in each strand as the real sheet showed that the band positions of the main ^{12}C- and ^{13}C-bands differed by only 0,2 cm^{-1} at most and that the ^{13}C-band shift from 100% 13C- to 10% 13C-content was the same.

IR spectra of the amide I vibration were calculated as previously described[15,16] but with the additional options to label all amide groups in a strand and to simulate a preference of $Aβ_{40}$ or $Aβ_{42}$ to form homogeneous aggregates in some calculations. The calculations assumed the same intrinsic wavenumber for all amide groups of a given isotope, nearest neighbor coupling constants from density functional theory calculations,[17] and transition dipole coupling between the remaining amide groups.

All amide groups in unlabeled strands were assigned a mass normalized diagonal force constant in the F matrix of 1.6138 mdyn $Å^{-1}$ u^{-1} corresponding to 1655 cm^{-1}. The respective numbers for labeled strands were 1.5429 mdyn $Å^{-1}$ u^{-1} and 1618 cm^{-1} and were calculated according to the mass effect on a C=O oscillator. The mass of the amide nitrogen atom has a negligible effect on the amide I frequency which was ignored in our considerations.

Non-nearest neighbor interactions were described by transition dipole coupling[18,19] using the following parameters: the dipole derivative of unlabeled amide groups had a magnitude of 3.2 D $Å^{-1}$ $u^{-1/2}$, was located on the C=O bond 0.868 Å away from the carbon atom,[20] and was oriented at an angle of 20° relative to the C=O bond towards the nitrogen atom. A transition dipole magnitude of approximately 0.32 D or the corresponding[21] value of 3.2 D $Å^{-1}$ $u^{-1/2}$ for the dipole derivative were found in experiments (0.33-0.35 D)[22,23] and in *ab initio* calculations (0.31-0.33 D)[24,25] with the model compound N-methyl acetamide in water. A dipole derivate between 3.1 and 3.2 D $Å^{-1}$ $u^{-1/2}$ was concluded for two β-sheet polypeptides[26] but higher values have also been reported (0.39 D corresponding to 3.9 D $Å^{-1}$ $u^{-1/2}$).[27] Angles of the transition dipole moment close to 20° have been measured for amide containing compounds[28,29] and the β-sheet protein silk fibroin[30] as well as computed in the above mentioned ab initio calculations.

The dipole derivative $\partial\mu/\partial q$ is calculated with respect to the normal coordinate q, which exhibits a square root dependency on the reduced mass m_r of the oscillator. For the labeled amide groups, the value of 3.2 D $Å^{-1}$ $u^{-1/2}$ for unlabeled amide groups was therefore multiplied with the square root of the ratio of the reduced masses $\{m_r(^{12}C{=}O)/m_r(^{13}C{=}O)\}^{1/2}$ for the unlabeled and labeled C=O oscillators.

In order to test the influence of structural distortions and mobility on the spectrum, we performed also one calculation in which the mass normalized diagonal force constants were statistically varied by at most ±1% and the non-diagonal force constants by at most ±10%. The former variation corresponds to a spread of the intrinsic frequencies within a 16 cm^{-1} interval and is close the effect of formation of a hydrogen bond the amide carbonyl oxygen.[31,32]

The statistical distribution of labeled and unlabeled strands in our sheets was modeled as described in the following. For each calculation, 3000 β-sheets with 6 strands each were generated. The total number of strands was partitioned into labeled and unlabeled strands according to one of the ^{13}C:^{12}C-ratios used in the experiments. These strands constituted the pool of strands used for the assignment of carbon isotopes to strands in the sheets. The number of ^{13}C- and ^{12}C-strands in the pool decreased as the assignment progressed from the first to the last strand of the 3000 sheets. For each strand, the probability for becoming labeled or unlabeled reflected the actual ^{13}C:^{12}C-ratio of the strands in the pool. After assigning carbon isotopes to all strands, the spectra of the 3000 sheets were calculated and averaged. We decided on the number of 3000 sheets because it provided good reproducibility of the averaged spectrum. Calculation of the average spectrum from 3000 sheets was repeated 20 times to generate 20 spectra for a given ^{13}C:^{12}C-ratio in order to assess the error in the obtained band positions.

Some of the calculations considered a preference for a given type of peptide to be next to itself in the aggregates. Since $Aβ_{40}$ and $Aβ_{42}$ had different carbon isotopes in our experiments, preference

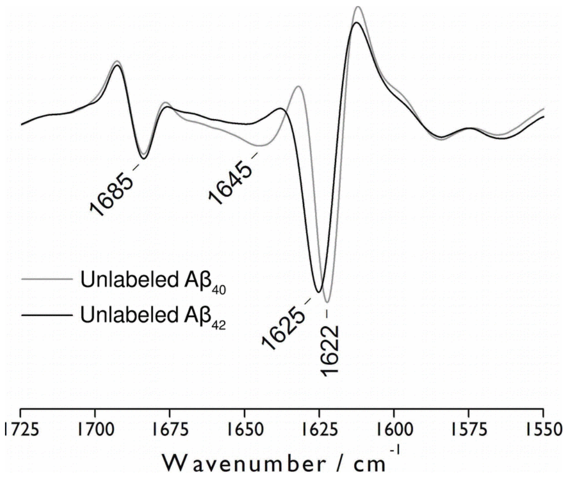

Fig. S1. Second derivative infrared spectra of oligomers obtained from $Aβ_{40}$ and $Aβ_{42}$ using unlabeled recombinant peptides. The vertical extensions between minimum and maximum are approximately 1.9 and 1.7 × 10^{-4} cm^2 for $Aβ_{40}$ and $Aβ_{42}$, respectively.

for homogeneous aggregates was modeled in our calculations by a preference of each carbon isotope to be next to a strand with the same isotope. This was done by considering an additional probability, the acceptance probability. Initially, the program assigns an isotope to the first strand as described above. It moves then to the next strand and assigns an isotope for this strand in the same way. Once this is done, it checks the isotope of the previous strand. If both strands contain the same isotope, then the isotope is accepted also for the actual strand. Otherwise, it is accepted or rejected according to a pre-defined acceptance probability. If the isotope is rejected, then the assignment of an isotope to the actual strand is repeated until an isotope for this strand is accepted. Then, the next strand is considered until all strands in all sheets are assigned.

Supplementary Results

Second derivative spectra of Aβ homo-aggregates from unlabeled peptides

Fig. S1 shows the second derivatives of infrared absorbance spectra of unlabeled $Aβ_{40}$ or $Aβ_{42}$ oligomers. The corresponding absorbance spectra after subtraction of residual solvent contributions are shown in Fig. S3. In second derivative spectra, negative bands correspond to component bands in the respective absorbance spectrum. Second derivatives are commonly used because component bands can be better distinguished and band positions more reliably determined than in absorbance spectra. In our case, they enable an evaluation without subtraction of residual solvent contributions in contrast to the processed absorbance spectra shown in Fig. S3. The reasons for this advantage of our second derivative spectra are twofold: (i) most of the solvent absorption is already subtracted in our original absorbance spectra due to the use of a buffer spectrum as background spectrum, and (ii) broad bands like those from the solvent are suppressed in second derivative spectra as compared to the sharper features of folded peptides.

The spectra of $Aβ_{40}$ and $Aβ_{42}$ oligomers show three main components in the amide I′ region (1700–1600 cm^{-1}) in ^2H_2O

buffer: (1) a sharp component at 1685 cm^{-1} assigned to β-sheets, (2) a weak and broad component between 1660 and 1640 cm^{-1} assigned to unstructured segments, and (3) a sharp and intense component at 1625-1622 cm^{-1} also assigned to β-sheets.[33–37] The latter dominates the spectrum which indicates a conformation rich in this secondary structure.

The high wavenumber band at 1685 cm^{-1} in Fig. S1 causes a pronounced shoulder in the absorbance spectra shown in Fig. S3. Such a spectral feature near 1695 cm^{-1} in $^{1}H_2O$ and in the dried state or at 1685 cm^{-1} in $^{2}H_2O$ is usually considered a marker band for antiparallel β-sheets.[38] There is considerable evidence for such an assignment including studies of amyloidogenic proteins[38] but it should be noted that a high wavenumber band also has been observed in spectra of some, but not all, β-helix proteins.[39] These proteins exhibit several bands in the amide I region, whereas our spectra show only the two distinct bands expected for antiparallel β-sheets. For these reasons, we assume an antiparallel β-sheet structure of our aggregates.

In order to quantify the relative contributions of the high and low wavenumber bands of β-sheets, we calculated the *β-sheet organizational index* from the baseline corrected, deconvoluted absorbance spectra according to Celej et al.[40] The index was 0.28 for Aβ$_{40}$ and 0.22 for Aβ$_{42}$ in line with previous findings for Aβ$_{42}$ oligomers.[40] The values are within the range found for antiparallel β-sheet proteins like avidin, concanavalin A and outer membrane porin OmpF (0.21-0.29).[40] Thus our spectra agree with those expected for proteins with a predominantly antiparallel organization of the β-sheets. Also other methods have indicated an at least in part antiparallel β-sheet structure of Aβ oligomers for Aβ fragments,[41–43] Aβ$_{40}$,[44] and Aβ$_{42}$.[45–49] Note however, that the conclusions of this work are independent from the structural interpretation of the data because very similar isotope dilution shifts were calculated for parallel and antiparallel sheets (compare Figs. 3 and S7).

Irrespective of the assignment of the high wavenumber band to a particular β-sheet architecture, it is considered a marker for oligomers and a key feature for discriminating between oligomers and amyloid fibrils.[38] This band is considerably weaker for Aβ fibril samples than for Aβ oligomer samples[1–5,50] and decreases in time resolved experiments concomitant with fibril formation.[1,50] Its presence correlates with the presence of spherical aggregates,[2–5,50] with weak fluorescence from the fibril marker thioflavin T,[1–3,5] with the inability of the aggregates to bind the amyloid specific antibody domain B10[3] and to alter the absorption of the amyloid stain Congo Red.[5] At an intermediate stage of the aggregation process, when the high wavenumber band is still present, the aggregates bind the oligomer-specific antibody A11, but not at a later stage, when the band is largely absent.[1]

In the following we compare the spectra of our preparations with those of the above cited oligomer studies. For this, we evaluated graphically the ratio of the absorbance values of the high wavenumber band and of the main β-sheet band. This ratio is related to the *β-sheet organizational index* but has the advantage that it can be evaluated from published spectra. The results are listed in Table S1. The ratios for our samples agree with or are larger than those for the previous Aβ$_{40}$ and Aβ$_{42}$ oligomer preparations. This shows that they contain predominantly oligomers.

The position of the main β-sheet at 1625-1622 cm^{-1} varies between the two peptide forms, with Aβ$_{40}$ showing a peak at

Table S1. Absorbance of the high wavenumber band divided by the absorbance of the main β-sheet band (H/M). The absorbance values were evaluated with respect to a baseline drawn between points near 1700 and near 1600 cm^{-1}. Our spectra were deconvoluted using the parameters of Cerf et al.[2] and our values are the averages and the standard errors of three independent experiments for each peptide.

Peptide	H/M	Reference
Unprocessed absorbance spectra		
Aβ$_{40}$	0.33 ± 0.01	This work
Aβ$_{40}$	0.27	Habicht et al. 2007[3]
Aβ$_{40}$	0.21	Breydo et al. 2016[4]
Aβ$_{42}$	0.24± 0.01	This work
Aβ$_{42}$	0.12	Eckert et al. 2008[5]
Deconvoluted absorbance spectra		
Aβ$_{40}$	0.32 ± 0.01	This work
Aβ$_{40}$ (0 h)	0.25	Sarroukh et al. 2011[1]
Aβ$_{40}$ (24 h)	0.10	Sarroukh et al. 2011[1]
Aβ$_{42}$	0.26 ± 0.01	This work
Aβ$_{42}$ (< 1 h)	0.22	Cerf et al. 2009[2]
Aβ$_{42}$ (24 h)	0.18	Cerf et al. 2009[2]

1622 cm^{-1} and Aβ$_{42}$ showing a peak near 1625 cm^{-1}. This small but distinctive feature indicates different backbone structures for Aβ$_{40}$ and Aβ$_{42}$. In general, a downshift of the main β-sheet band can be due to (i) more planar β-sheets, (ii) β-sheet sandwiches with less layers, or larger inter-layer distance, or larger angle of rotation between the layers, (iii) more strands per sheet, or (iv) stronger hydrogen bonding to the backbone carbonyls.[15,27,33,51,52] The latter explanation seems to be unlikely. Stronger hydrogen bonding should also downshift the high wavenumber band of antiparallel β-sheets, which is not observed. Explanation (iii) - more strands per sheet - does not seem appropriate in the light of our gel electrophoresis results (see below) which show that the Aβ$_{42}$ oligomers include larger species than the Aβ$_{40}$ oligomers. However, we cannot exclude this explanation because larger species may be composed of smaller sheets that are separated enough to be vibrationally decoupled. Such a separation will lead to an upshift of the main β-sheet band and the argument has been used before to explain the upshift of a β-sheet band upon nucleotide dissociation from the sarcoplasmic reticulum Ca^{2+}-ATPase.[53] In conclusion, differences in β-sheet size, distortion, or layer architecture are likely causes of the spectral differences between Aβ$_{40}$ and Aβ$_{42}$. Such changes leave the band position of the high wavenumber β-sheet band relatively unaffected[27,54] (for β-sheet layers this statement is only true when the layer distance exceeds ~7Å[15]). In line with the above interpretation, we observe the high wavenumber band at the same position in both types of oligomers.

We note also that the 1625-1622 cm^{-1} band is broader for Aβ$_{42}$ than for Aβ$_{40}$, which indicates the contribution of sheets with different structures to the Aβ$_{42}$ spectrum, either because this is an inherent property of individual Aβ$_{42}$ oligomers, or because of the wider distribution of oligomer sizes in the Aβ$_{42}$ preparation detected by gel electrophoresis (see below).

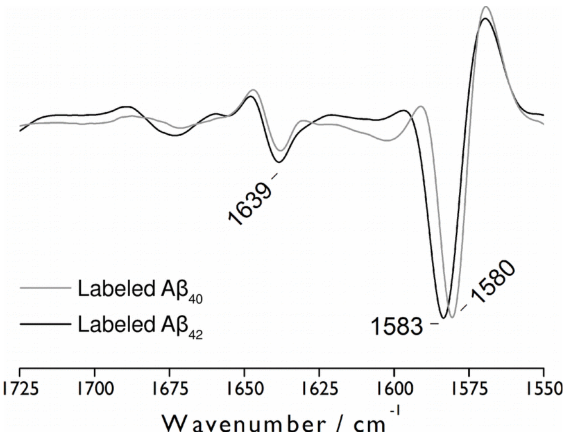

Fig. S2. Second derivative infrared spectra of oligomers obtained from Aβ$_{40}$ and Aβ$_{42}$ using ^{13}C,^{15}N-labeled recombinant peptides. The vertical extensions between minimum and maximum are approximately 1.7 and 2.0 × 10^{-4} cm^{2} for Aβ$_{40}$ and Aβ$_{42}$, respectively.

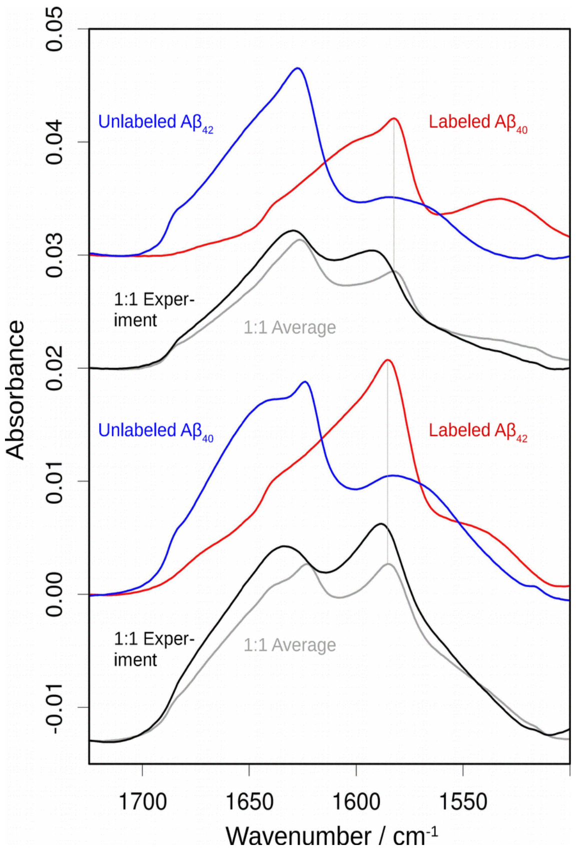

Fig. S3. IR absorbance spectra of Aβ oligomers corresponding to the second derivative spectra shown in Figs. S1, S2 and in Fig. 1 of the main text. The spectra are offset for a clearer presentation. Spectra from unlabeled peptides are shown in blue and those from labeled peptides in red. "1:1 Average" labels the average spectrum of the blue and the red spectrum shown above it. "1:1 Experiment" labels the respective experimental spectrum of a 1:1 mixture. The vertical lines indicate the ^{13}C-band positions of the homo-oligomers.

The spectra of the Aβ oligomers in our preparations are consistent with those reported in the literature.[2,3,38,55] Most previous studies used non-deuterated samples (often dried) and therefore find the absorbance bands at slightly higher wavenumbers. In contrast, our samples were measured in ^{2}H$_{2}$O solution, which causes an exchange of the amide hydrogen for deuterium and in consequence a downshift of the amide I band. Our band positions are similar to those of Aβ$_{42}$ interacting with membranes in ^{2}H$_{2}$O (1685 and 1623 cm^{-1})[56] and to those of Aβ$_{40}$ dried from ^{2}H$_{2}$O solution (1685 and 1624 cm^{-1}).[57] Interestingly, in our previous study of Aβ$_{40}$ in ^{2}H$_{2}$O,[55] incubated for the longer time of 15 h, the main band position was slightly below 1620 cm^{-1} and 35% broader. The high wavenumber band (near 1685 cm^{-1}) was less intense relative to the main β-sheet band (by a factor of nearly 5 in second derivative spectra) and had a similar width as in the present study. Considering both the different widths of the main β-sheet bands and the different intensities of the high wavenumber bands, it is concluded that longer incubation considerably reduces the band area of the high wavenumber band compared to the band area of the main band. This can be interpreted as a much smaller content of antiparallel β-sheets and a larger content of parallel β-sheet structures upon prolonged incubation in line with previous findings.[1,50,58]

Second derivative spectra of Aβ homo-oligomers from labeled peptides

The second derivative spectra of oligomers obtained from uniformly ^{13}C,^{15}N-labeled Aβ$_{40}$ and Aβ$_{42}$ are shown in Fig. S2 and their corresponding absorbance spectra in Fig. S3. Their band pattern is similar to the corresponding unlabeled peptides, although all amide I' bands are shifted to lower wavenumbers. The high wavenumber band is now located at 1639 cm^{-1}, while the main β-sheet band is located at 1580 cm^{-1} in Aβ$_{40}$ oligomers, and at 1583 cm^{-1} in Aβ$_{42}$ oligomers. A comparison of the spectra obtained from labeled and unlabeled oligomers confirms the expectation that labeled Aβ$_{40}$ and Aβ$_{42}$ oligomers adopt similar, if not identical structures as oligomers of the corresponding unlabeled peptides.

The spectral shifts originate from the larger mass of heavy isotopes incorporated in the labeled peptides (^{13}C and ^{15}N), leading to an increase in the reduced mass of the oscillators and, hence, a decrease in their vibrational frequencies. Because the amide I mode is largely dominated by the C=O stretching vibration (~80%), most of the shift is caused by the ^{13}C-isotope. The ^{15}N-isotope contributes to the shift to a much smaller extent (1-2 cm^{-1})[59,60] in the form of a minor contribution of the C-N stretching and C-C-N bending vibrations to the amide I mode.[18] ^{15}N-labeling has therefore little impact on our results and the reason for using doubly labeled peptides was merely their commercial availability. Our ^{13}C-band position for Aβ$_{42}$ is close to that obtained by drying Aβ$_{42}$ from an HFIP solution and submerging the film in ^{2}H$_{2}$O (1585 cm^{-1}).[61] However, the authors of that study do not report a high wavenumber component and conclude that the structure consists of parallel β-sheets.

IR absorbance spectra of Aβ oligomers

IR absorbance spectra of labeled and unlabeled Aβ$_{40}$ and Aβ$_{42}$ homo-oligomers are shown in Fig. S3 as well as spectra of the experimental 1:1 mixtures and the respective calculated average spectrum of the two homo-oligomer spectra. In line with the second derivative spectra, the absorbance spectra of the experimental 1:1 mixtures are different from the calculated

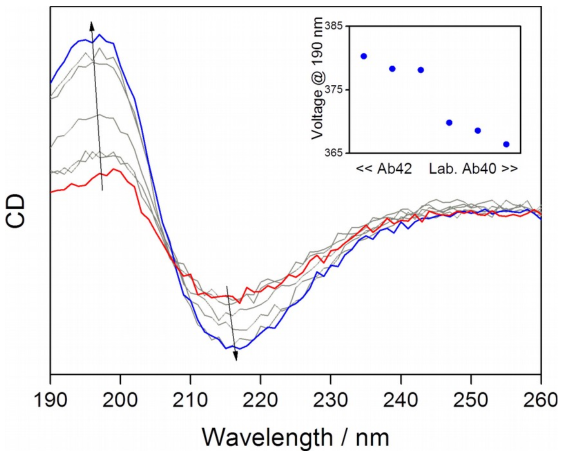

Fig. S4. CD spectra of Aβ_{42} (blue), Aβ_{40} (red) oligomers, and mixed oligomers (grey). The detector voltage at 190 nm measured for each spectrum is shown in the inset.

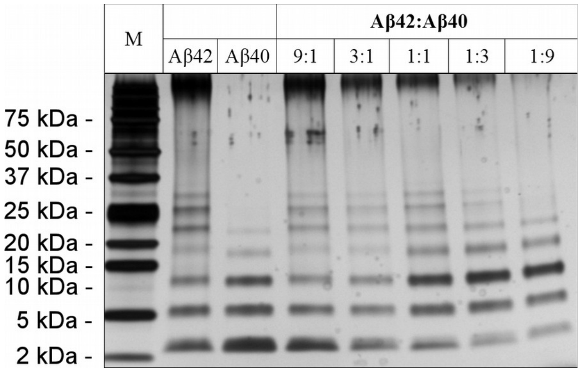

Fig. S5. SDS-PAGE of photo-crosslinked Aβ_{42} oligomers, Aβ_{40} oligomers and mixed oligomers.

average spectra. Bands in the experimental spectra appear broader and the band positions are shifted which indicates the formation of mixed oligomers.

Composition effect on oligomer size distribution

Circular dichrosim (CD) spectra of pure Aβ_{42} and Aβ_{40} oligomers as well as of mixed oligomers are shown in Fig. S4. All spectra indicate a predominant β-sheet composition, as denoted by the maxima and minima at 197 and 216 nm, respectively. Although all spectra were recorded at the same molar concentration (200 µM), Aβ_{42} oligomers exhibit a larger β-sheet signal than Aβ_{40} oligomers and this signal correlates with the relative amount of Aβ_{42} of in the mixed oligomers. This is in line with the lower random coil content of Aβ_{42}-rich oligomers detected with IR spectroscopy.

In the CD experiments, the detector voltage increased with increasing Aβ_{42} ratio. This experimental parameter is often used as a monitor for light scattering, and its increase might correlate with an increase in particle size.

To test whether the relative amounts of Aβ_{42} and Aβ_{40} in the oligomers corresponded to different oligomer sizes, the oligomers characterized in Fig. S4 were photo-crosslinked and their size analyzed by SDS-PAGE. The results, shown in Fig. S5, indicate that pure Aβ_{42} and Aβ_{40} oligomers have different sizes. In the case of

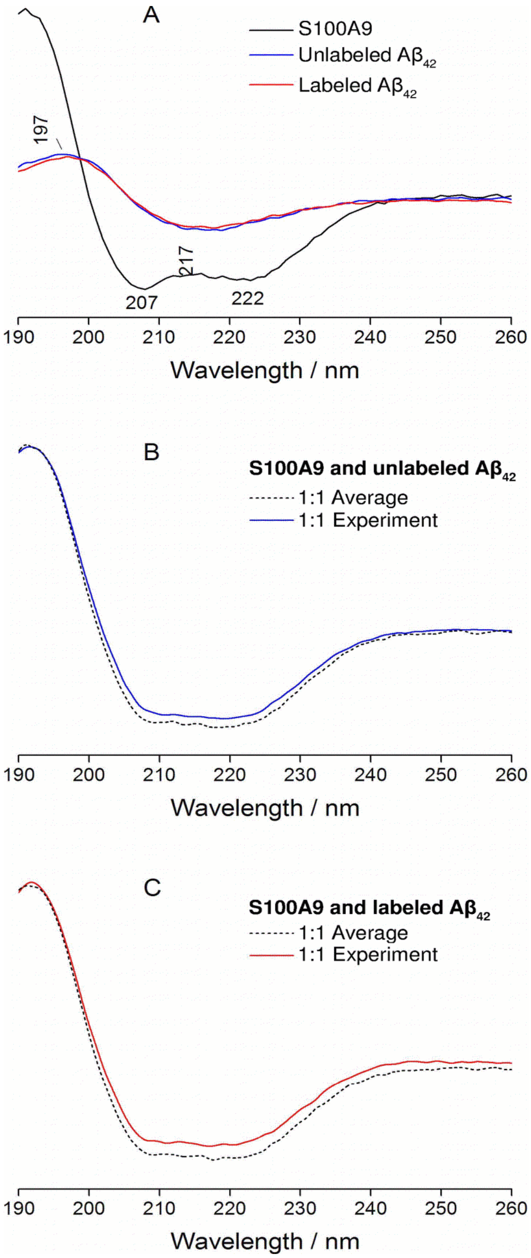

Fig. S6. CD spectra of unlabeled and labeled Aβ_{42} in the presence of S100A9. (A) CD spectra of unlabeled (blue) and labeled (red) Aβ_{42}, together with that of S100A9 (black). (B) and (C) Theoretical and experimental CD spectra of the Aβ_{42} and S100A9 1:1 mixture.

pure Aβ_{42} oligomers, dimers and trimers coexisted with medium-molecular weight (4- to 7-mers) and large species some of which had weights larger than 250 kDa. Pure Aβ_{40} oligomers, however, mainly existed as low-molecular weight species (up to tetramer), and no sign of large species was observed. In mixed oligomers, the increase in the relative amount of Aβ_{42} corresponded to an increase in medium-molecular weight and high molecular weight species. These results are in line with the enhanced light scattering of Aβ_{42} containing oligomers, which was inferred from the detector voltage in the CD experiments.

An additional property, that could not be concluded from either CD or IR data, was the preferential organization of mixed

oligomers containing small amounts of Aβ$_{42}$ into trimeric structures, as can be observed in the last three lanes of the gel. The trimeric species was not the dominant species in either pure Aβ$_{42}$ or Aβ$_{40}$ oligomers, and therefore represents a preferential organization of Aβ$_{40}$-rich mixed oligomers. The highest relative abundance for this species was observed in 1:3 and in the 1:9 oligomers, which is in line with the previous finding of predominantly smaller oligomers for similar Aβ$_{42}$:Aβ$_{40}$ ratios.[62] These ratios correspond to the most common Aβ$_{42}$:Aβ$_{40}$ ratio found in patients affected by Alzheimer's disease, therefore representing a potential link between oligomer size and appearance of the disease.[62–64]

CD spectra of Aβ$_{42}$ oligomers in the presence of S100A9

IR spectra of Aβ42 oligomer formation in the presence of S100A9 are shown in panels B and D of Fig. 6 of the main text. As a complement to the IR data, CD spectra were also recorded immediately after the IR measurements. The results are shown in Fig. S6. The CD spectrum of S100A9 (panel A) shows the typical profile of all-α proteins, namely two negative bands at 207 and 222 nm and a positive band at 193 nm. The CD spectra of unlabeled and labeled Aβ42 oligomers are indistinguishable from each other. They both show a negative band at 217 nm, and a positive band at 197 nm. This is in full agreement with the spectra of β-sheet proteins. The experimental and calculated spectra of the mixtures (panels B and C) are virtually superimposable onto each other, considering a small baseline drift. Thus CD spectroscopy does not detect a conformational change due to interaction between Aβ42 and S100A9 and is insensitive to the subtle changes detected by IR spectroscopy discussed in the main text.

Calculated amide I spectra of parallel β-sheets with mixed isotope composition

Fig. S7 shows calculated amide I spectra of parallel β-sheets which correspond to those for antiparallel sheets shown in Fig. 3 of the main text. Isotopic dilution causes a qualitatively similar band shift of the ^{13}C-band as for the antiparallel sheet. The shift is 14 cm^{-1} from 10% to 100% ^{13}C for the parallel sheet and 17 cm^{-1} for the antiparallel sheet.

Sensitivity of the approach to detect a deviation from random mixing

Our isotope mixing experiments revealed shifts of the ^{13}C-band position with changing ^{13}C:^{12}C ratio which are shown in Fig. 2 of the main text. Two of the ^{13}C-band position curves were obtained for randomly mixed oligomers (gray lines), the two others characterized Aβ$_{42}$:Aβ$_{40}$ mixtures (black lines). The former superimpose well on the latter, indicating that these two peptides form common β-sheets which contain a random mixture of Aβ$_{40}$ and Aβ$_{42}$ strands. One could however argue, that our method is simply insensitive to a deviation from random mixture. To scrutinize such an objection, we simulated ^{13}C-band shifts under the condition that a given strand prefers to be adjacent to a strand with the same isotope. In the context of the Aβ$_{42}$:Aβ$_{40}$ mixing experiment this means a preference of each peptide to be adjacent to its own kind in the β-sheets, e.g. Aβ$_{40}$ favors to be next to Aβ$_{40}$. We will explore in the following the expected consequences of a deviation from random mixing for the ^{13}C-band position curves. We will then compare the simulated results to the experimental results in Fig. 2.

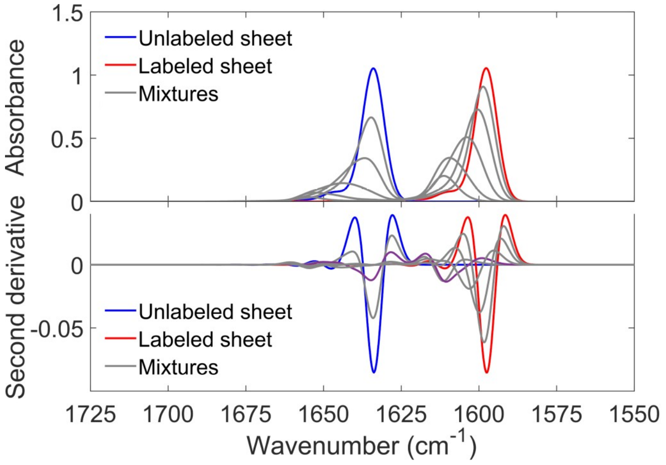

Fig. S7. Calculated amide I spectra for a parallel β-sheet with 6 strands and 10 residues per strand (9 complete amide groups). The blue and red spectra were calculated for entirely unlabeled and labeled sheets, respectively. The gray spectra were calculated for different molar fractions of ^{13}C-peptides (0.1, 0.25, 0.5, 0.75, 0.9). Each simulated spectrum is the average of the spectra from 60000 sheets with a statistical distribution of labeled and unlabeled strands at a given ^{13}C:^{12}C-ratio. ^{13}C-enrichment gradually shifts the ^{13}C-band from 1611.3 to 1597.7 cm^{-1}. The ^{12}C band position for the entirely unlabeled sheet is 1634.0 cm^{-1}.

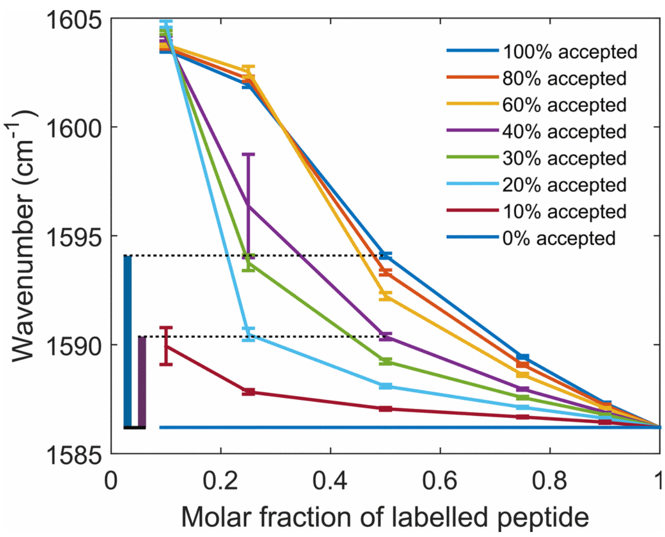

Fig. S8. Calculated position of the ^{13}C-absorption band of antiparallel β-sheets with 6 strands and 10 residues for each strand. Plotted is the average band position of 20 spectra each of which was the average of the spectra of 3000 sheets. The error bars indicate the standard deviation calculated from the 20 repetitions. The vertical bars on the left illustrate the ^{13}C-band shift at 100% (blue) and 40% (purple) acceptance probability when half of the peptides are labeled.

Fig. S8 shows calculated ^{13}C-band positions for an antiparallel β-sheet. The calculations modeled a preference of Aβ$_{40}$ and Aβ$_{42}$ for forming homogeneous oligomers. This was implemented in the program for spectrum calculation by an acceptance probability of a given strand to accept a neighboring strand in the sheet with a different isotope. 100% acceptance probability means that mixing is random. In contrast, 0% acceptance probability generates only

isotopically pure sheets, modeling a situation where $A\beta_{40}$ and $A\beta_{42}$ do not mix in the oligomers. In this case, the band positions for the ^{12}C- and the ^{13}C-band do not shift, when the ^{13}C:^{12}C-ratio is changed, because all sheets are isotopically pure. Consequently, the respective curve for the ^{13}C-band position is flat in Fig. S8. All other curves lie between this curve and the curve for 100% acceptance. The curves for low acceptance probabilities exhibit a steep slope at low ^{13}C:^{12}C-ratios, because ^{12}C is only incorporated into ^{13}C-sheets when there is a large excess of ^{12}C. The largest absolute deviation of curves with preference for pure oligomers from the curve for random mixing is observed at intermediate ^{13}C:^{12}C-ratios. For example at a ^{13}C:^{12}C-ratio of 0.5, an acceptance probability of 40% decreases the shift of the ^{13}C-band to 54% (purple bar in Fig. S8) of the value for pure sheets (blue bar in Fig. S8). This is in contrast to the experimental ^{13}C-band position curves shown in Fig. 2. Those for the $A\beta_{42}$:$A\beta_{40}$ mixtures (black lines) deviate very little from the curves for random mixing (gray lines), in particular at intermediate ^{13}C:^{12}C-ratios. The deviation is considerably less than for the simulated curve with 40% acceptance probability. Therefore we conclude that the acceptance probability in our experiments is between 40% and 100%.

The property acceptance probability is related to the composition of the β-sheets. In order to give an impression of the composition of the sheets at the two limiting acceptance probability values of 40% and 100%, Table S2 lists the composition of the simulated sheets for the two cases. From the values at 40% acceptance, we conclude that a 1:1 mixture leads to simulated 6-stranded sheets, of which 55% contain 2-4 strands of either isotope/peptide. The respective percentage for completely random mixing is 78%. Accordingly in our experiments, a 1:1 monomer mixture leads to $A\beta_{42}$:$A\beta_{40}$ oligomers of which a majority contains between one third and two thirds of either peptide. Thus the simulations support the conclusion from the experiments that $A\beta_{40}$ and $A\beta_{42}$ monomers form common β-sheets, where they are mixed randomly or nearly randomly.

Table S2. Isotopic composition of 6-stranded antiparallel β-sheets. The numbers in the table give the percentage of sheets with a certain number of labeled strands at a given molar fraction of labeled strands. The top number in regular type in each cell states the fraction of sheets at 100% acceptance probability, the bottom number in italics type the fraction at 40% acceptance probability.

Number of ^{13}C-strands per sheet	^{13}C:^{12}C-ratio						
	0	0.1	0.25	0.5	0.75	0.9	1
	Percentage of sheets						
**0**	100<br>*100*	53.2<br>*61.9*	18.1<br>*32.4*	1.4<br>*9.4*	0.0<br>*1.3*	0.0<br>*0.1*	0.0<br>*0.0*
**1**	*0.0*<br>*0.0*	35.4<br>*23.5*	35.0<br>*25.2*	9.4<br>*13.2*	0.5<br>*3.0*	0.0<br>*0.4*	0.0<br>*0.0*
**2**	*0.0*<br>*0.0*	9.9<br>*9.5*	29.9<br>*19.1*	23.8<br>*17.8*	3.4<br>*6.7*	0.1<br>*1.1*	*0.0*<br>*0.0*
**3**	*0.0*<br>*0.0*	1.4<br>*3.5*	13.2<br>*12.3*	31.3<br>*19.2*	13.0<br>*12.3*	1.3<br>*3.4*	*0.0*<br>*0.0*
**4**	*0.0*<br>*0.0*	0.1<br>*1.1*	3.4<br>*6.7*	23.2<br>*17.7*	29.8<br>*19.3*	9.8<br>*9.5*	*0.0*<br>*0.0*
**5**	*0.0*<br>*0.0*	0.0<br>*0.4*	0.4<br>*3.0*	9.4<br>*13.4*	35.5<br>*25.1*	36.0<br>*23.6*	*0.0*<br>*0.0*
**6**	*0.0*<br>*0.0*	0.0<br>*0.1*	0.0<br>*1.3*	1.6<br>*9.3*	17.8<br>*32.4*	52.8<br>*61.8*	100<br>*100*

References

1 1R. Sarroukh, E. Cerf, S. Derclaye, Y. F. Dufrêne, E. Goormaghtigh, J.-M. Ruysschaert and V. Raussens, *Cell. Mol. Life Sci.*, 2011, **68**, 1429–38.

2 E. Cerf, R. Sarroukh, S. Tamamizu-Kato, L. Breydo, S. Derclaye, Y. F. Dufrêne, V. Narayanaswami, E. Goormaghtigh, J.-M. Ruysschaert and V. Raussens, *Biochem. J.*, 2009, **421**, 415–23.

3 G. Habicht, C. Haupt, R. P. Friedrich, P. Hortschansky, C. Sachse, J. Meinhardt, K. Wieligmann, G. P. Gellermann, M. Brodhun, J. Götz, K.-J. Halbhuber, C. Röcken, U. Horn and M. Fändrich, *Proc. Natl. Acad. Sci. U. S. A.*, 2007, **104**, 19232–7.

4 L. Breydo, D. Kurouski, S. Rasool, S. Milton, J. W. Wu, V. N. Uversky, I. K. Lednev and C. G. Glabe, *Biochem. Biophys. Res. Commun.*, 2016, **477**, 700–705.

5 A. Eckert, S. Hauptmann, I. Scherping, J. Meinhardt, V. Rhein, S. Dröse, U. Brandt, M. Fändrich, W. E. Müller and J. Götz, *J. Mol. Med.*, 2008, **86**, 1255–1267.

6 T. Vogl, N. Leukert, K. Barczyk, K. Strupat and J. Roth, *Biochim. Biophys. Acta - Mol. Cell Res.*, 2006, **1763**, 1298–1306.

7 M. Baldassarre and A. Barth, *Analyst*, 2014, **139**, 5393–9.

8 M. Baldassarre, C. Li, N. Eremina, E. Goormaghtigh and A. Barth, *Molecules*, 2015, **20**, 12599–12622.

9 G. Bitan, A. Lomakin and D. B. Teplow, *J. Biol. Chem.*, 2001, **276**, 35176–84.

10 G. Bitan, *Methods Enzymol.*, 2006, **413**, 217–236.

11 R. Fraser and T. MacRae, ed. R. Fraser, Academic Press, 1973, pp. 218–246.

12 L. Pauling and R. B. Corey, *Proc. Natl. Acad. Sci. USA*, 1953, **39**, 253–256.

13 J. D. Dunitz, *Angew. Chemie Int. Ed.*, 2001, **40**, 4167–4173.

14 C. E. Chivers, A. L. Koner, E. D. Lowe and M. Howarth, *Biochem. J.*, 2011, **435**, 55–63.

15 E.-L. Karjalainen, H. K. Ravi and A. Barth, *J. Phys. Chem. B*, 2011, **115**, 749–57.

16 E.-L. Karjalainen, T. Ersmark and A. Barth, *J. Phys. Chem. B*, 2012, **116**, 4831–4842.

17 T. la Cour Jansen, A. G. Dijkstra, T. M. Watson, J. D. Hirst and J. Knoester, *J. Chem. Phys.*, 2006, **125**, 44312.

18 S. Krimm and J. Bandekar, *Adv. Prot. Chem.*, 1986, **38**, 181–367.

19 H. Torii and M. Tasumi, in *Infrared spectroscopy of biomolecules*, eds. H. H. Mantsch and D. Chapman, Wiley-Liss, New York, 1996, pp. 1–18.

20 W. H. Moore and S. Krimm, *Biopolymers*, 1976, **15**, 2439–2464.

21 T. C. Cheam and S. Krimm, *Chem. Phys. Lett.*, 1984, **107**, 613–616.

22 J. Kubelka and T. A. Keiderling, *J. Phys. Chem. A*, 2001, **105**, 10922–10928.

23 M. Grechko and M. T. Zanni, *J. Chem. Phys.*, 2012, **137**, 184202.

24 T. la Cour Jansen, J. Knoester and C. Jansen, *J. Chem. Phys.*, 2006, **124**, 44502.

25 J. Wang, *Phys. Chem. Chem. Phys.*, 2009, 5310–5322.

26 T. C. Cheam and S. Krimm, *J. Chem. Phys.*, 1985, **82**, 1631–1641.

27 Y. N. Chirgadze and N. A. Nevskaya, *Biopolymers*, 1976, **15**, 607–25.

28 I. Sandeman, *Proc. R. Soc. A Math. Phys. Eng. Sci.*, 1955, **232**, 105–113.

29 E. M. Bradbury and A. Elliott, *Spectrochim. Acta*, 1963, **19**,

995–1012.
30 E. Suzuki, *Spectrochim. Acta Part A Mol. Spectrosc.*, 1967, **23**, 2303–2308.
31 H. Torii, T. Tatsumi, T. Kanazawa and M. Tasumi, *J. Phys. Chem. B*, 1998, **102**, 309–314.
32 H. Torii, T. Tatsumi and M. Tasumi, *J. Raman Spectrosc.*, 1998, **29**, 537–546.
33 A. Barth, *Biochim. Biophys. Acta*, 2007, **1767**, 1073–101.
34 E. Goormaghtigh, V. Cabiaux and J. M. Ruysschaert, *Subcell. Biochem.*, 1994, **23**, 405–50.
35 M. Jackson and H. H. Mantsch, *Crit. Rev. Biochem. Mol. Biol.*, 1995, **30**, 95–120.
36 J. L. R. Arrondo and F. M. Goñi, *Prog. Biophys. Mol. Biol.*, 1999, **72**, 367–405.
37 H. Fabian and W. Mäntele, in *Handbook of vibrational spectroscopy*, eds. J. M. Chalmers and P. Griffiths, John Wiley & Sons, Chichester, 2002, pp. 3399–3426.
38 R. Sarroukh, E. Goormaghtigh, J.-M. Ruysschaert and V. Raussens, *Biochim. Biophys. Acta - Biomembr.*, 2013, **1828**, 2328–2338.
39 R. Khurana and A. L. Fink, *Biophys. J.*, 2000, **78**, 994–1000.
40 M. S. Celej, R. Sarroukh, E. Goormaghtigh, G. D. Fidelio, J.-M. Ruysschaert and V. Raussens, *Biochem. J.*, 2012, **443**, 719–726.
41 R. K. Spencer, H. Li and J. S. Nowick, *J. Am. Chem. Soc.*, 2014, **136**, 5595–5598.
42 T. D. Do, N. E. LaPointe, R. Nelson, P. Krotee, E. Y. Hayden, B. Ulrich, S. Quan, S. C. Feinstein, D. B. Teplow, D. Eisenberg, J.-E. Shea and M. T. Bowers, *J. Am. Chem. Soc.*, 2016, **138**, 549–557.
43 V. A. Streltsov, J. N. Varghese, C. L. Masters and S. D. Nuttall, *J. Neurosci.*, 2011, **31**, 1419–26.
44 B. Chandra, D. Bhowmik, B. K. Maity, K. R. Mote, D. Dhara, R. Venkatramani, S. Maiti and P. K. Madhu, *Biophys. J.*, 2017, **113**, 805–816.
45 L. Gu, C. Liu and Z. Guo, *J. Biol. Chem.*, 2013, **288**, 18673–18683.
46 L. Yu, R. Edalji, J. E. Harlan, T. F. Holzman, A. P. Lopez, B. Labkovsky, H. Hillen, S. Barghorn, U. Ebert, P. L. Richardson, L. Miesbauer, L. Solomon, D. Bartley, K. Walter, R. W. Johnson, P. J. Hajduk and E. T. Olejniczak, *Biochemistry*, 2009, **48**, 1870–7.
47 C. Lendel, M. Bjerring, A. Dubnovitsky, R. T. Kelly, A. Filippov, O. N. Antzutkin, N. C. Nielsen and T. Härd, *Angew. Chemie Int. Ed.*, 2014, **53**, 12756–12760.
48 M. Ahmed, J. Davis, D. Aucoin, T. Sato, S. Ahuja, S. Aimoto, J. I. Elliott, W. E. Van Nostrand and S. O. Smith, *Nat. Struct. Mol. Biol.*, 2010, **17**, 561–7.
49 L. Gu, C. Liu, J. C. Stroud, S. Ngo, L. Jiang and Z. Guo, *J. Biol. Chem.*, 2014, **289**, 27300–27313.
50 A. Itkin, V. Dupres, Y. F. Dufrêne, B. Bechinger, J.-M. Ruysschaert and V. Raussens, *PLoS One*, 2011, **6**, e18250.
51 H. Torii and M. Tasumi, *J. Chem. Phys.*, 1992, **96**, 3379–3387.
52 J. Kubelka and T. A. Keiderling, *J. Am. Chem. Soc.*, 2001, **123**, 12048–12058.
53 M. Liu and A. Barth, *J. Biol. Chem.*, 2004, **279**, 49902–9.
54 P. Bour and T. A. Keiderling, *J. Mol. Struct.*, 2004, **675**, 95–105.
55 A. Abelein, J. Jarvet, A. Barth, A. Gräslund and J. Danielsson, *J. Am. Chem. Soc.*, 2016, **138**, 6893–6902.
56 V. Koppaka and P. H. Axelsen, *Biochemistry*, 2000, **39**, 10011–10016.
57 C. Paul and P. H. Axelsen, *J. Am. Chem. Soc.*, 2005, **127**, 5754–5.
58 S. Chimon, M. A. Shaibat, C. R. Jones, D. C. Calero, B. Aizezi and Y. Ishii, *Nat. Struct. Mol. Biol.*, 2007, **14**, 1157–64.
59 P. I. Haris, G. T. Robillard, A. A. Van Dijk and D. Chapman, *Biochemistry*, 1992, **31**, 6279–6284.
60 G. Cuevas, V. Renugopalakrishnan, G. Madrid and A. T. Hagler, *Phys. Chem. Chem. Phys.*, 2002, **4**, 1490–1499.
61 J. O. Matos, G. Goldblatt, J. Jeon, B. Chen and S. A. Tatulian, *J. Phys. Chem. B*, 2014, **118**, 5637–5643.
62 K. Pauwels, T. L. Williams, K. L. Morris, W. Jonckheere, A. Vandersteen, G. Kelly, J. Schymkowitz, F. Rousseau, A. Pastore, L. C. Serpell and K. Broersen, *J. Biol. Chem.*, 2012, **287**, 5650–5660.
63 P. Lewczuk, N. Lelental, P. Spitzer, J. M. Maler and J. Kornhuber, *J. Alzheimers. Dis.*, 2015, **43**, 183–91.
64 J. Wiltfang, H. Esselmann, M. Bibl, M. Hüll, H. Hampel, H. Kessler, L. Frölich, J. Schröder, O. Peters, F. Jessen, C. Luckhaus, R. Perneczky, H. Jahn, M. Fiszer, J. M. Maler, R. Zimmermann, R. Bruckmoser, J. Kornhuber and P. Lewczuk, *J. Neurochem.*, 2007, **101**, 1053–1059.
